# Deep Bayesian active learning using in-memory computing hardware

**DOI:** 10.1038/s43588-024-00744-y

**Published:** 2024-12-23

**Authors:** Yudeng Lin, Bin Gao, Jianshi Tang, Qingtian Zhang, He Qian, Huaqiang Wu

**Affiliations:** School of Integrated Circuits, Beijing National Research Center for Information Science and Technology, Tsinghua University, Beijing, China

**Keywords:** Electronic devices, Computer science

## Abstract

Labeling data is a time-consuming, labor-intensive and costly procedure for many artificial intelligence tasks. Deep Bayesian active learning (DBAL) boosts labeling efficiency exponentially, substantially reducing costs. However, DBAL demands high-bandwidth data transfer and probabilistic computing, posing great challenges for conventional deterministic hardware. Here we propose a memristor stochastic gradient Langevin dynamics in situ learning method that uses the stochastic of memristor modulation to learn efficiency, enabling DBAL within the computation-in-memory (CIM) framework. To prove the feasibility and effectiveness of the proposed method, we implemented in-memory DBAL on a memristor-based stochastic CIM system and successfully demonstrated a robot’s skill learning task. The inherent stochastic characteristics of memristors allow a four-layer memristor Bayesian deep neural network to efficiently identify and learn from uncertain samples. Compared with cutting-edge conventional complementary metal-oxide-semiconductor-based hardware implementation, the stochastic CIM system achieves a remarkable 44% boost in speed and could conserve 153 times more energy.

## Abstract (web summary)

This study introduces an in-memory deep Bayesian active learning framework that uses the stochastic properties of memristors for in situ probabilistic computations. This framework can greatly improve the efficiency and speed of artificial intelligence learning tasks, as demonstrated with a robot skill-learning task.

## Main

**Fig. 1:**
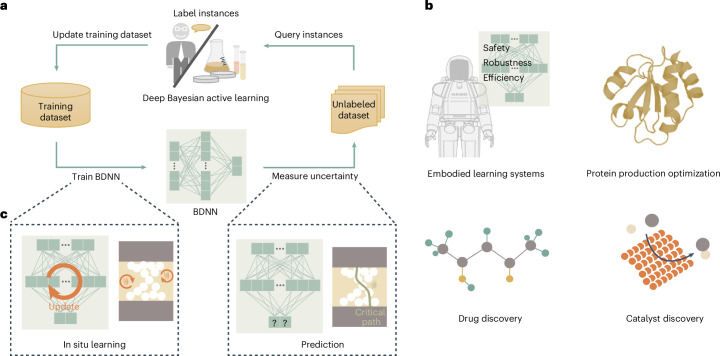
DBAL. **a**, The process of DBAL. In DBAL, a BDNN is trained on a small amount of data (the initial training set) and then measures uncertainty of unlabeled data points. The highest value instances are selected on the basis of uncertainty to ask an external oracle for a label. An oracle (often a human expert) labels the selected instances. These are added to the training set, and the BDNN is retrained on the updated training set. This process is then repeated, with the training set increasing in size over time. **b**, Applications of DBAL. Embodied learning systems, catalyst discovery, drug discovery and protein production optimization are often limited by wet lab labor and cost as well as the lack of convenient computational tools. **c**, Memristor-based neuromorphic hardware can enables fast and power-efficient DBAL by exploiting device stochastic characteristics. Panel **b** reproduced with permission from: top left, ref. ^40^ under a Creative Commons license CC BY 4.0; top right, ref. ^41^ under a Creative Commons license CC BY 4.0.

Deep learning has made substantial progress in a variety of complex artificial intelligence (AI) tasks, primarily due to the availability of enormous labeled datasets. However, labeling data is a time-consuming, labor-intensive and costly procedure, particularly in professional domains requiring substantial expertise^1^. For instance, real-world robots struggle to swiftly evaluate and label skill-specific actions owing to the time and resource overhead of executions and resetting experimental scenarios^2,3^. In contrast, deep Bayesian active learning (DBAL) can overcome the labeling bottleneck (Fig. 1a). Labeling efficiency for learning is crucial, especially for edge learning, which poses particularly strict demands on power consumption, latency and adaptivity^4^. DBAL acquires and labels informative data to learn, achieving high-quality learning using as few labeled data as possible. It can exponentially improve labeling efficiency, leading to substantial cost savings. Therefore, DBAL emerges as a compelling learning solution in many AI-enabled scenarios where labels are limited or expensive to obtain (Fig. 1b), such as embodied learning systems^2,5^, catalyst discovery^6,7^, drug discovery^8,9^ and protein production optimization^10–12^.

DBAL is typically implemented with conventional deterministic von-Neumann hardware with complementary metal-oxide-semiconductor (CMOS)-based chips^13,14^, such as software-based DBAL in previous work^8^. Intensive vector–matrix multiplication (VMM) in training often causes data shuffling between the processor and memory, leading to substantial latency and energy consumption^15^. Furthermore, DBAL involves numerous random variables with Gaussian distributions to capture uncertainty^16^. Generating Gaussian random numbers for weight updates during training introduces great computational latency and energy consumption, surpassing that of VMM owing to its complexity^17^. In contrast, probabilistic computation based on memristor arrays not only eliminates this extensive data movement during VMM computation but also uses the intrinsic randomness properties of memristors to efficiently generate random numbers^18–20^ (Fig. 1c). The memristor-based computation-in-memory (CIM) technologies store the weights in nanodevices of crossbar arrays^21–26^. Hence, the VMM in situ computation can be realized with only one parallel read operation based on Ohm’s law and Kirchhoff’s current law^27^. Meanwhile, the random motion of ions in memristors gives the conductance random properties, enabling efficient mimicry of random number generation through read or program operations. Research shows that memristor arrays can effectively implement probabilistic AI algorithms^28–32^.

However, the iterative learning process of DBAL relies heavily on accurate uncertainty capture, which helps select informative samples for labeling. Misjudging uncertainty may hinder the identification of useful unlabeled data, increase labeling costs and potentially fail the learning task. Therefore, DBAL not only requires learning high-level feature extraction for predictions such as traditional deep learning but also needs to learn the dispersion of massive probability weights to capture uncertainty. This feature involving random number generation makes the hardware overhead of DBAL considerable. Hence, there are still challenges to implement DBAL using memristor. First, it is challenging to realize the learning from scratch of memristor Bayesian deep neural network (BDNN) in DBAL, owing to nonlinearity conductance modulation. Second, the stochastic behaviors of memristor conductance should enable the network to capture uncertainty in learning process and also present uncertainty of prediction. Third, it should be addressed that the excessive conductance stochasticity has negative effects on the learning methods.

In this work, we propose an in-memory DBAL framework. An initial deployed memristor BDNN, obtained by ex situ training, learns in situ iteratively with selected data to capture uncertainty. This hardware-based framework allows BDNN to achieve efficient learning. Second, we propose a memristor stochastic gradient Langevin dynamics (mSGLD) in situ learning method that uses the stochasticity of device. Using a single modulation pulse can generate Gaussian random numbers for weight updating. The proposed method will transit to an end of learning, where the stochastic of device reading is used. Third, we propose a smooth transition method to mitigate the impact of excessive conductance stochasticity on learning. These proposed methods allow the memristor BDNN to efficiently capture and present uncertainty during learning and prediction. Finally, based on these methods, we implement in-memory DBAL on a memristor-based stochastic CIM system and successfully demonstrate a robot’s skill learning task with minimal labeled samples. The task shows a great speed and energy efficiency boost compared with conventional CMOS hardware. Our work presents an efficient DBAL implementation using a memristor-based system and demonstrates efficient probabilistic computations fundamental to Bayesian methods.

## Results

### Synapse weights stochastic characteristics

**Fig. 2:**
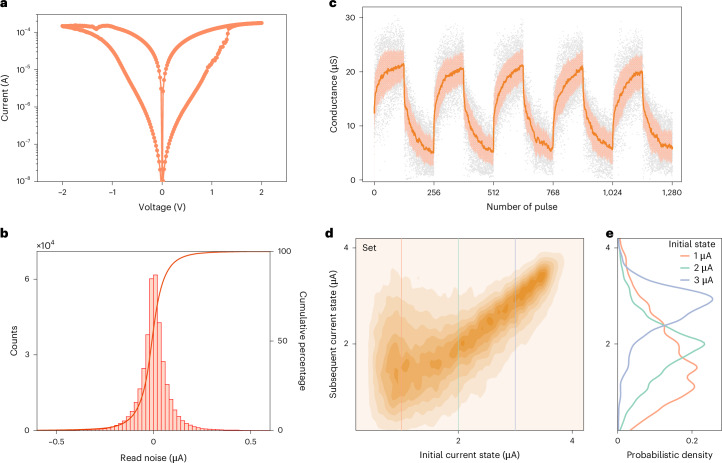
Stochastic characteristics of memristors. **a**, A typical measured *I*–*V* curve of a single 1T1R cell for a quasi-d.c. sweep. **b**, The probability density of the read noise in the 3.3 μA current state at read voltage *V*_read_ = 0.2 V, measured over 1,000 read cycles across individual arrays. **c**, Typical analog switching behaviors of memristors under identical pulse trains. The dark lines represent the average values of the conductance, the light colors fill the regions between plus and minus one standard deviation, and the gray dots represent measured data. **d**, The statistical distribution of the conductance transition from their initial states to states for devices in a 4K chip, under a single set pulse with constant-amplitude voltage *V*_set_ = 2.0 V. The gate voltage of the transistor is *V*_t_ = 1.25 V. The current state is measured at read voltage *V*_read_ = 0.2 V. **e**, Probability density curves of the conductance transition at three initial states (*I*_read_ = 1 μA, 2 μA and 3 μA). Each curve corresponds to a profile along the vertical lines shown in **d**. Source data

**Extended Data Fig. 1:**
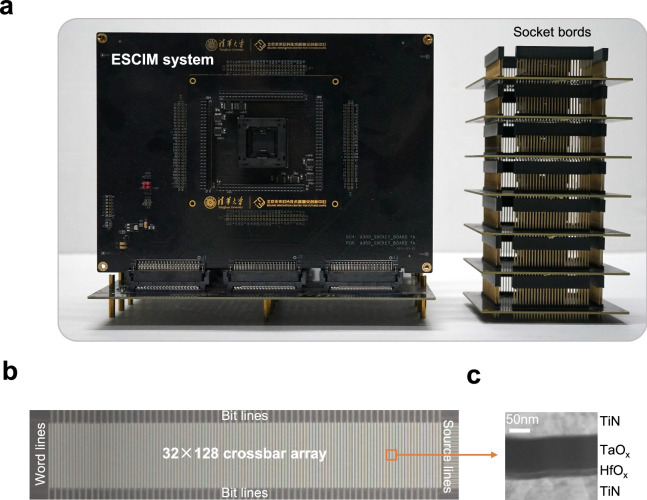
ESCIM system. **a**, Photograph of the ESCIM system. The ESCIM system contains 64 ADCs, FPGAs for control and communication, and a power supply integrated at the PCB level. The 8 memristor chips can be stacked by using the socket board shown on the right side of the picture. **b**, Optical microscope image of 4 K array. The 32 SLs were perpendicular to the 128 BLs and parallel to the 32 WLs. **c**, Memristors adopted the TiN/TaO_x_/HfO_x_/TiN material stack.

To implement in-memory DBAL, we used an expandable stochastic CIM computing (ESCIM) system (Extended Data Fig. 1). The ESICM system, expandable through stacking, integrated three memristor chips in this study. The memristor device adopted the TiN/TaO_*x*_/HfO_*x*_/TiN material stack. Figure 2a depicts the current–voltage (*I*–*V*) curves of the 1-transistor-1-memristor (1T1R) cell, which are smooth and symmetrical. Thanks to TaO_*x*_, which serves as a thermally enhanced layer^33^, the multilevel characteristics of the memristor are improved.

**Extended Data Fig. 2:**
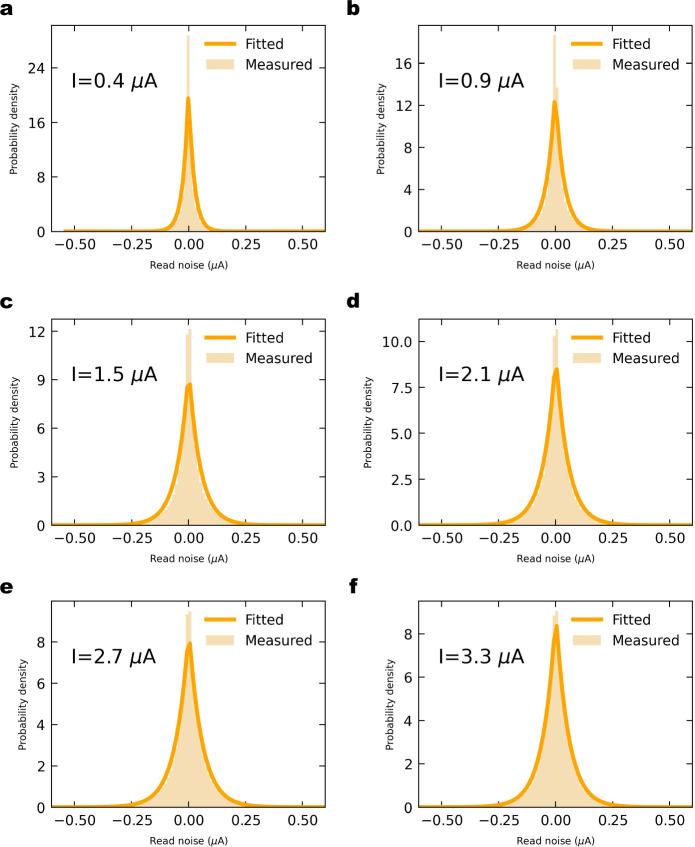
Probability density of the read noise for around 2 K memristors in a specific conductance state. **a**–**f**, Each plot shows the empirical distribution of the read noise superimposed with the corresponding fitted Laplace distribution. Source data

**Extended Data Fig. 3:**
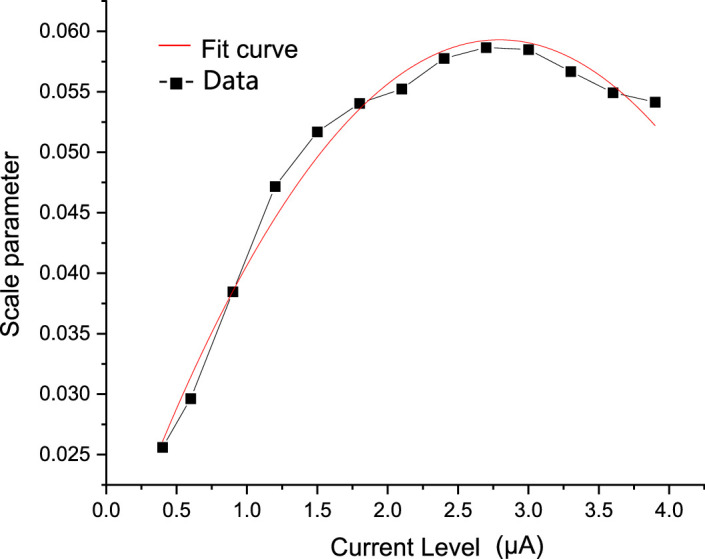
Memristors in different current states have distinct random fluctuation characteristics. The vertical axis represents the scale parameter of the Laplace distribution fitting function, which reflects the dispersion of the distribution. The larger the value, the more dispersed the distribution is. Source data

**Extended Data Fig. 4:**
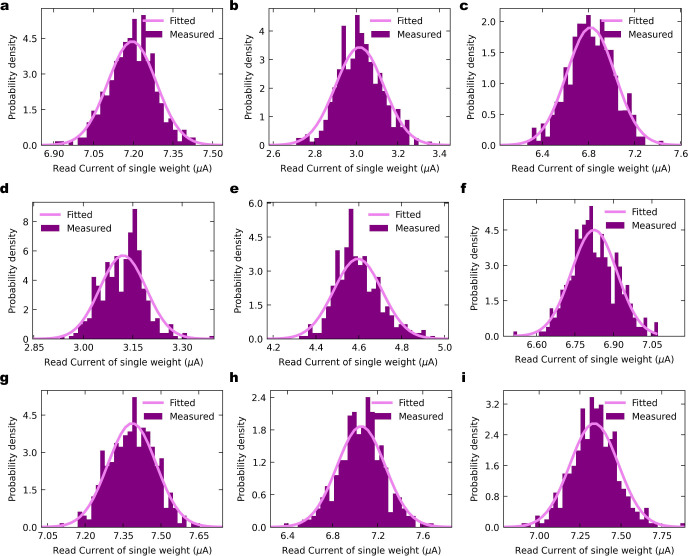
Typical single-weight distribution fitted with a normal distribution. **a**–**i**, Nine typical single-weight distributions. Each distribution shape is obtained from 360-times read results. The Gaussian weight is produced using three devices in the ESCIM system. Source data

**Extended Data Fig. 5:**
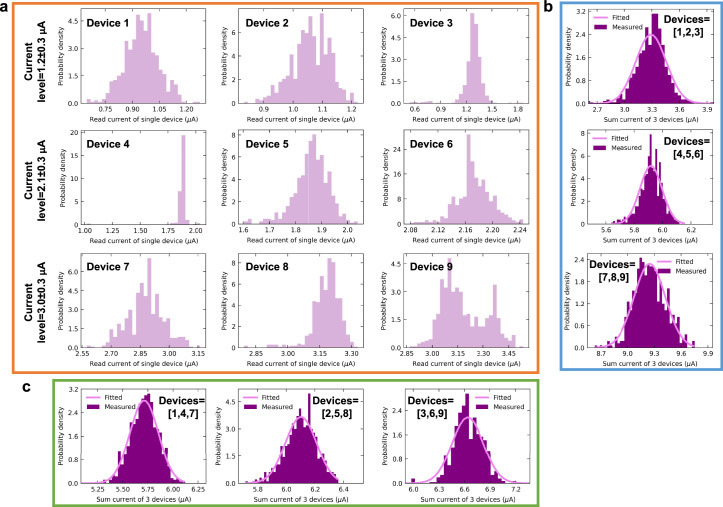
Integration of device-to-device variability and read-to-read (or cycle-cycle) variability in Gaussian weight. **a**, Each histogram represents the probability density of the read current for a single device, labelled Device 1 through Device 9. Current level of devices [1,2,3], [4,5,6], and [7,8,9] are 1.2, 2.1 and 3.0 ± 0.3 μA, respectively. **b**, Fitted and measured probability density functions for the sum of currents from three grouped devices: [1,2,3], [4,5,6], and [7,8,9]. Three devices are at the same current levels. **c**, Fitted and measured probability density functions for the sum of currents from different groupings of three devices: [1,4,7], [2,5,8], and [3,6,9]. Three devices are at the different current levels. Source data

To analyze the stochastic characteristics of the memristor, we measured conductance variations during the reading and modulation process. On the one hand, the fluctuation data collected from the reading test can be modeled using a double exponential distribution (Fig. 2b). Memristors in different current states have distinct random fluctuation characteristics (Extended Data Figs. 2 and 3). The measured results of read noise align well with the previous reports of current fluctuation behaviors in HfO_*x*_-based memristor devices^34,35^ (Supplementary Note 1). By adjusting the memristor to an appropriate current state, various probability distributions can be obtained (‘In situ sampling via reading memristors’ section in Methods). Such unique stochastic characteristics can facilitate in situ random number generation by reading the current. According to the Lindeberg–Feller central limit theorem (Supplementary Note 2), the Gaussian distribution can be realized by accumulating the currents of multiple memristors (Extended Data Fig. 4). Hence, the ESCIM system can efficiently perform both in situ Gaussian random number generation and in-memory computation, integrating the devices and cycles variabilities of memristors (Extended Data Fig. 5 and Supplementary Note 3).

**Extended Data Fig. 6:**
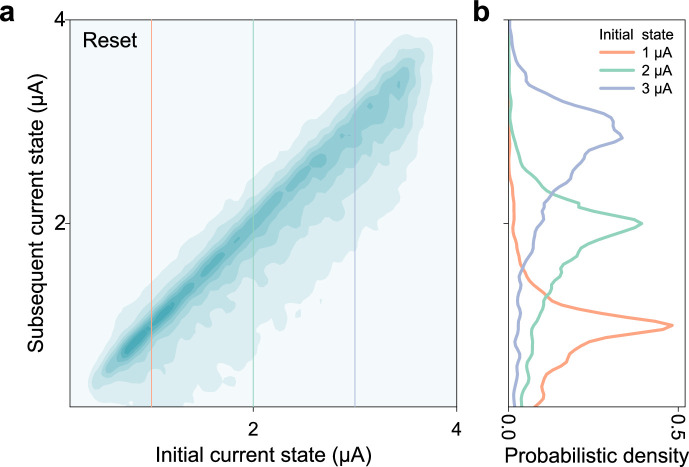
The conductance transition in the case of reset operation. **a**, Statistical distribution of the conductance transition from their initial states to states for devices in a 4 K chip, under a single reset pulse with constant-amplitude voltage *V*_*reset*_ = 1.95 V. The gate voltage of transistor is *V*_*t*_ = 2.55 V. The current state is measured at read voltage *V*_*read*_ = 0.2 V. **b**, Probability density curves of the conductance transition at three initial states (*I*_*read*_ = 1μA, 2μA and 3μA). Each curve corresponds to a profile along the vertical lines shown in **a**. Source data

On the other hand, memristors also have random fluctuations during the conductance modulation process. Under identical-amplitude voltage pulse modulation, the memristor exhibits continuous bidirectional resistive switching characteristics (Fig. 2c). Meanwhile, random migration of oxygen ions within the resistive layer can cause variations impacting the conductance of different devices, even within the same device across cycles. To quantitatively analyze these inherent stochastic characteristics, we measured conductance transitions (‘Measurements of the conductance transition’ section in Methods). We applied a constant-amplitude voltage pulse to the 1T1R cells, prompting the memristors to transition from initial to subsequent conductance states. Figure 2d shows the conductance transition distribution during the set operation. While the subsequent conductance generally increases during a set operation, decreases can occur due to random oxygen ion migration. Furthermore, the spread of the transition distribution varies depending on the initial conductance state. Figure 2e shows the transition probability curves for three different initial conductance states, clearly showing an alignment with a Gaussian distribution. In addition, the conductance transition in the case of reset operation also exhibits a similar Gaussian distribution (Extended Data Fig. 6). Therefore, the operation of a single pulse during a memristor’s conductance modulation can be modeled as drawing a random number from a Gaussian distribution.

### In-memory DBAL

**Fig. 3:**
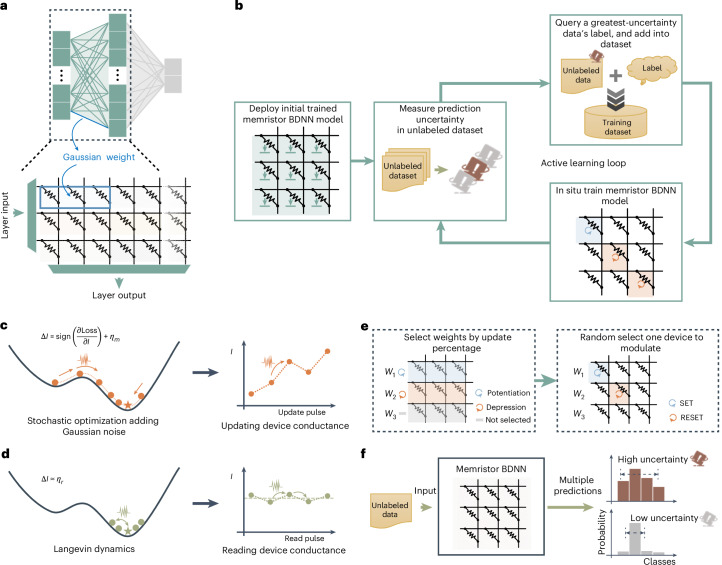
In-memory DBAL and mSGLD in situ learning method. **a**, Realization of a Gaussian weight of a BDNN in memristor crossbar array. Read currents accumulated from three devices act as one Gaussian weight. **b**, The proposed in-memory Bayesian active learning flow chart. **c**, The initial phase of the proposed mSGLD. The conductance of the memristor is updated according to the sign of the gradient to mimic an effective stochastic gradient learning algorithm. The added Gaussian noise *η*_*m*_ can be realized by the random fluctuations of conductance modulation process. **d**, The latter phase of the proposed mSGLD. The network optimization process came into a flat minimum of the loss function, and the magnitude of the gradient diminished. Gaussian noise of the total read current dominates, thus mimicking the Langevin dynamic MH process. **e**, Realization of the weight updating. Left: a certain percentage of weights with large gradient magnitude is selected to be updated, and this update ratio keeps decreasing with the number of training iterations. Right: one of the three devices of these selected weights is randomly chosen for modulation to realize the weight update. **f**, By making multiple predictions, the prediction distribution can be efficiently obtained and, thus, the uncertainty can be calculated.

Consistent with the Lindeberg–Feller central limit theorem, Gaussian weights in a BDNN can be simulated using read currents from multiple devices (Fig. 3a). Within our implementation strategy, a Gaussian weight is produced using three devices in the ESCIM system. As shown in Fig. 3b, we proposed the in-memory DBAL framework building on a memristor BDNN (Supplementary Note 4).

**Extended Data Fig. 7:**
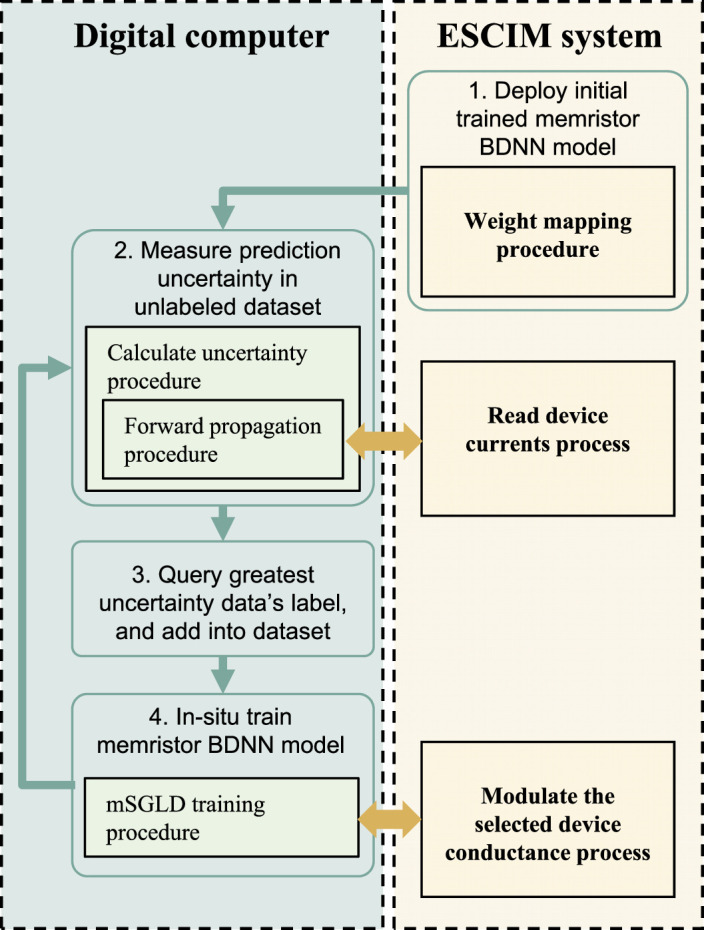
Flowchart for DBAL integrated a digital computer and ESCIM system. In the robot’s skill learning task demonstration, the process involves deploying the initial model, measuring prediction uncertainty, querying data labels, and in-situ training, with interactions between weight mapping, device current reading, and conductance modulation implemented on ESCIM system.

The proposed in-memory DBAL framework integrates a digital computer and our ESCIM system (Extended Data Fig. 7). First, an initial memristor BDNN model is deployed on memristor crossbar arrays (for the pseudocode, refer to Supplementary Fig. 1). This model’s weights are obtained by ex situ training on a digital computer using a small initial training dataset. The read noise model and conductance modulation model are used during this process, enabling the network to learn weight distributions that better fit the integrated memristor arrays (‘Stochastic models of memristors’ section in Methods). Next, the deployed memristor BDNN predicts the classes of data in the unlabeled dataset (Supplementary Fig. 2), and calculates prediction uncertainty (Supplementary Fig. 3). This process involves fully turning on the transistors in the crossbar array, applying the read voltage to the source line (SL) of the device row by row and sensing the fluctuating read current that flows through the virtual ground BL by an analog-to-digital converter (ADC). The network prediction, due to the weight’s stochasticity introduced by the memristor cells’ variabilities, is a distribution reflecting the variability in read currents rather than a single deterministic value (‘Uncertainty quantification’ section in Methods). Consequently, we can use the multiple prediction outputs from the network to derive a prediction distribution, thereby calculating the prediction uncertainty. Subsequently, based on the prediction uncertainty of the samples in the unlabeled dataset, we select a data sample with the highest uncertainty to query for its label and incorporate this sample and the queried label into the training dataset. A data sample with the highest uncertainty typically contains more information, and its label is generally the most beneficial for enhancing the network’s classification performance. Finally, using the updated training dataset, which includes the original training data and the newly added samples, the memristor BDNN performs in situ learning. After in situ learning, the network continues to calculate uncertainty, select high-uncertainty samples and retrain until performance expectations are met or label queries are exhausted.

In the process of active learning, the in situ learning step is crucial. Given the limited quantity of samples in the training dataset, inadequate in situ learning capacity could lead to a network with deficient classification capabilities. This might hinder the quantification of uncertainty, thereby challenging the identification of useful unlabeled data samples. Even after multiple rounds of active learning, the network performance might still not meet the anticipated standards.

### In situ learning to capture uncertainty

To accurately capture uncertainty in DBAL’s iterative learning, we proposed an in situ learning method using the stochastic property of the conductance modulation process (Supplementary Fig. 4). The method is an improvement based on the stochastic gradient Langevin dynamics (SGLD) algorithm^16^. The weight parameter update in the SGLD algorithm is very straightforward: it takes the gradient step of traditional training algorithms^36^ and adds an amount of Gaussian noise. The training process of SGLD includes two phases. In the initial phase, the gradient will be dominant and the algorithm will mimic an efficient stochastic gradient algorithm. As step sizes decay with the number of training iterations, in the latter phase, the injected Gaussian noise will be dominant and, therefore, the algorithm will mimic the Langevin dynamic Metropolis–Hastings (MH) algorithm. As the number of training iterations increases, the algorithm will smoothly transit between the two phases. Using the SGLD method allows the weight parameters to capture parameter uncertainty and not just collapse to the maximum a posteriori solution.

Based on the stochastic nature of memristors, we improved the SGLD algorithm using sign backpropagation, namely, mSGLD. The stochastic fluctuation under constant-amplitude pulses can also be considered as random number generation. In the initial phase of mSGLD, we calculated the gradient of the memristor conductance $\frac{\partial {{\mathrm{Loss}}}}{\partial I}$ (‘mSGLD training method’ section in Methods) and then updated the value of the memristor weight based on the sign of the gradient to mimic an effective stochastic gradient learning algorithm

1 $$\Delta I={\rm{sign}}\left(\frac{\partial {{\mathrm{Loss}}}}{\partial I}\right)+{\eta }_{{\mathrm{m}}}.$$

Since the transition probability of the conductance during modulation is Gaussian distribution, the added Gaussian noise *η*_m_ can be realized by the random fluctuations inherent in the device (Fig. 3c). Therefore, the actual update operation of the device on the memristor array is

2 $${\rm{sign}}\left(\frac{\partial {\mathrm{Loss}}}{\partial I}\right)=\left\{\begin{array}{rcl}1 & \to & {\rm{Set}}\,{\rm{device}}\\ -1 & \to & {\rm{Reset}}\,{\rm{device}}.\end{array}\right.$$

That is, if the sign of the gradient of a device is positive, a set operation is performed on the device; if it is negative, a reset operation is performed.

**Extended Data Fig. 8:**
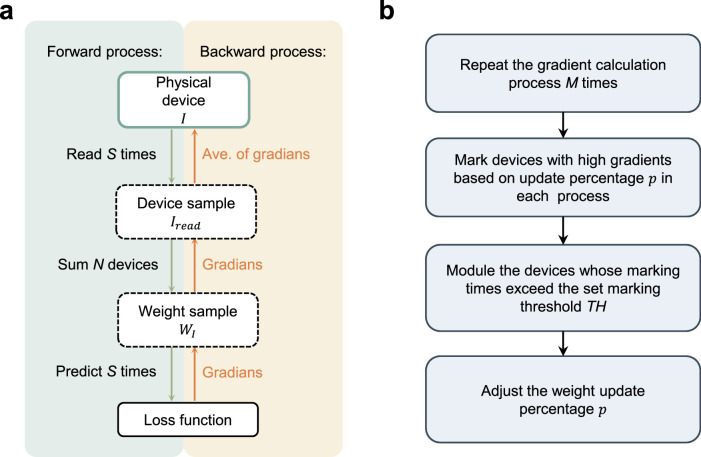
Computation of gradient and device selected method. **a**, The gradient $\frac{\partial Loss}{\partial I}$ is computed by backpropagation method. **b**, For smooth transition into the latter phase of mSGLD, we choose a progressively smaller weight update percentage *p* to only update the key weights during iterative training.

In the later phase of mSGLD, with more training iterations, the memristor network’s classification performance improves. As it reaches a flat loss function minimum, the gradient diminishes and injected Gaussian noise becomes dominant (Fig. 3d). For smooth transition between the two phases, we proposed a smooth transition method. In this method, we update a decreasing percentage of weights with large gradients as training iterations increase (‘mSGLD training method’ section in Methods and Extended Data Fig. 8). Then, for the selected weights, one of three devices is randomly chosen for modulation to update the weight (Fig. 3e). Therefore, as the number of training iterations increases, the training ends and the number of updated weights decreases to a small amount. Finally, the Gaussian noise of the total read current dominates, thus mimicking the Langevin dynamic MH process. By gradually decreasing the weight update ratio, the smooth transition between phases also reduces the negative impact of excessive conductance stochasticity, stabilizing the network learning process. We also thoroughly discuss management of noise in mSGLD (Supplementary Figs. 5 and 6), using regular SGD instead of mSGLD (Supplementary Fig. 7), and the impact of binarizing the gradient (Supplementary Fig. 8) in a BDAL simulation experiment based on a Modified National Institute of Standards and Technology (MNIST) dataset classification task. The results show the effectiveness and resilience of our proposed mSGLD method (Supplementary Note 5).

Our proposed in situ learning algorithms leverage stochastic characteristics in the reading and conductance modulation process for Gaussian random number generation during network prediction and learning. In BDNN learning, weights are updated with gradient values added Gaussian noise, allowing Bayesian parameter uncertainty capture via the in situ mSGLD method. In BDNN prediction, Gaussian weights are sampled and computed through VMM with the input vector. Memristor Gaussian weights in crossbar arrays enable efficient weight sampling and VMM with a single read operation. Multiple predictions yield the prediction distribution and calculate uncertainty (Fig. 3f).

### Robot’s skill learning

**Fig. 4:**
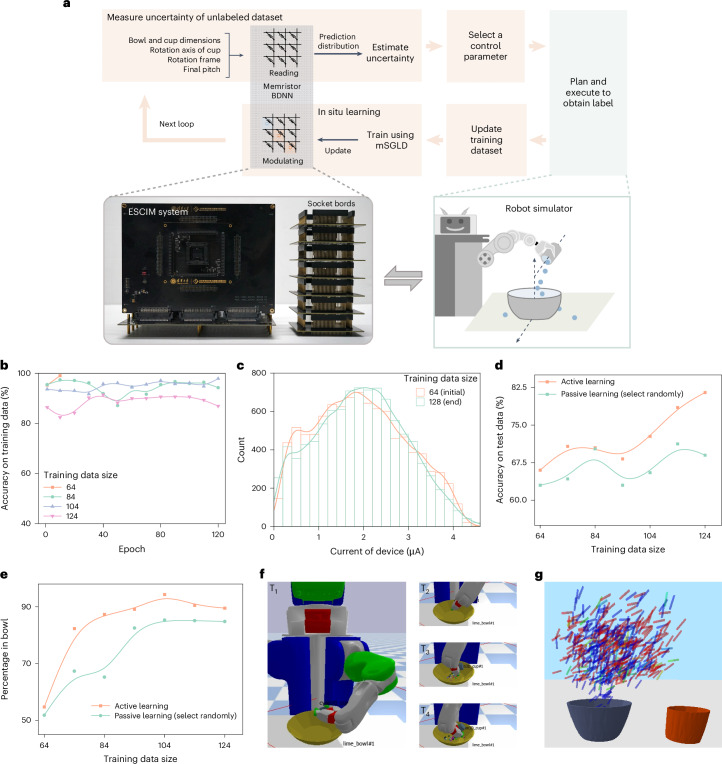
Robot’s skill learning and experimental results. **a**, A schematic illustration of robot’s pouring skill learning task using in-memory DBAL. **b**, The evolution of the accuracy on training data of the memristor BDNN with each epoch on 64, 84, 104 and 124 training dataset sizes. **c**, Histogram (bar) and distribution curve (line) of memristor conductance state at the initial and end of the active learning loop. Measured at read voltage *V*_read_ = 0.2 V. **d**, The classification accuracy on the test dataset of the active learning method and the passive learning method. Active learning has an advantage compared with passive learning, which randomly selects the samples to be queried. **e**, The learning performance of active and passive learning methods. We used the percentage of pouring the contents of the cup into the bowl as a performance metric. **f**, Visualization of the active learning results. The robot is pouring the beads from the cup into the bowl. T_1_, T_2_, T_3_ and T_4_ represent sequential time points in the process of pouring. **g**, Visualization of the final cup position for 500 pour valid control parameters. It visualizes the most confident (low uncertainty) prediction of the memristor BDNN by coloring small values in red and large values in blue. Source data

To demonstrate the applicability of the proposed methods, we performed a demonstration in a robot’s skill learning task (Fig. 4a). In this learning task (‘Robot’s pouring skill learning task and robot simulator’ section in Methods), the robot is equipped with a set of basic abilities such as locomotion and basic object manipulation. The robot needs to build on these foundations by training a BDNN model to acquire pouring high-level skills. However, the labeled data required to learn the skill are very difficult, time-consuming or expensive to obtain due to resource overhead and time overhead. Therefore, the robot needs to efficiently learn the pouring skill with as few labeled samples or attempts as possible, thus minimizing the experiments time, labor and cost for obtaining labeled data.

The expected effect of the pouring action is to pour the contents of a cup into a bowl. We are interested in learning under what constraints the execution of this action will transfer a sufficiently large portion of the initial contents of the cup into the target bowl. These constraints for a pour are the context parameters (the bowl and cup dimensions) and control parameters that the robot can choose (the axis of rotation, the cup rotation frame and the final pitch). To execute successful pouring action, we used a memristor BDNN to predict whether the execution of the pouring action will be successful or not under different control parameters and then proceed to the next step of plan and execution based on the control parameters that have a higher probability of success (Fig. 4a). Thus, our main objective is to train a BDNN to achieve high accuracy and action effectiveness using as few labeled samples as possible, through the proposed active learning methods.

We implemented an active learning task using an 11 × 50 × 50 × 2 memristor BDNN (‘Experiment system setup’ section in Methods), balancing hardware complexity and network performance (Supplementary Note 6). The BDNN was trained on a digital computer using a 64-sample dataset, then deployed to the ESCIM system. In the active learning loop (Fig. 4a), the memristor BDNN predicts and estimates uncertainties of 10,000 unlabeled constraints, queries the label of the most uncertain constraint and adds it to the training set. The memristor BDNN then uses the updated dataset for 110 epoch iterations of in situ learning via the proposed mSGLD method. If training accuracy reaches 98% or more in several iterations, in situ learning ends early. This loop is repeated 64 times, cumulatively querying 64 constraints, resulting in a final training dataset of 128 samples.

We successfully demonstrated the in-memory active learning process of this task using the ESCIM system. We used a digital computer to set up a three-dimensional (3D) tabletop simulator with a robot and control DBAL loop, as shown by the orange and light green parts of Fig. 4a (‘Experiment system setup’ section in Methods). Supplementary Fig. 9 shows the pseudocode for the robot’s skill learning task using the in-memory DBAL framework. Our active learning method was extensively evaluated using the simulator. The ESCIM system, connected to the digital computer, read and modulated memristor arrays’ conductance during BDNN prediction and in situ learning, as shown by the gray parts in Fig. 4a. Supplementary Note 7 provides additional technical details on the robot’s skill learning process. Figure 4b depicts the memristor BDNN’s training classification accuracy evolution across four different training dataset sizes, indicating high accuracy across all networks. Notably, with 64 training samples, in situ learning stops early due to the smaller data size’s reduced complexity and noise. The memristor conductance state distribution of the initial and end of the active learning loop is shown in Fig. 4c. We also measured the passive learning method for comparison, which randomly selects samples for querying instead of on the basis of prediction uncertainty. Figure 4d shows the classification accuracy of active and passive learning method with unseen testing data. Increasing training data size generally enhances the model’s generalization and test accuracy. It can be seen that initial network classification accuracies of both methods are similar. However, as query samples increase, active learning outperforms passive learning, improving by about 13%. We also analyze the impact of cycle-to-cycle variability on network’s performance over time (Supplementary Note 8). The network maintains stable performance over time, with accuracy levels similar to those post in situ learning (Supplementary Fig. 10). The reason for the stability may be that the BDNN can inherently tolerate certain weights’ variations caused by cycle-to-cycle variability. Furthermore, we compared the learning performance of active and passive learning on the pouring skill task (Fig. 4e), with results showing that active learning outperforms passive learning with the same number of query samples.

We visualized the process of the robot pouring the beads from the cup into the bowl using active learning, as shown in Fig. 4f and Supplementary Movie 1. We also visualized the final tipping angle of the cup at the end of the execution of the pouring action by the robot. Figure 4g shows a dataset of pouring control parameters for a single bowl and cup pair by showing the final position of the red cup. We find that the memristor BDNN learns that either the cup has a larger pitch and is located directly above the bowl or the cup has a smaller pitch and is located slightly to the right of the bowl. This suggests that the memristor BDNN is capturing intuitively relevant information for a successful pour. These results show that the proposed methods could realize efficient in-memory DBAL.

**Extended Data Fig. 9:**
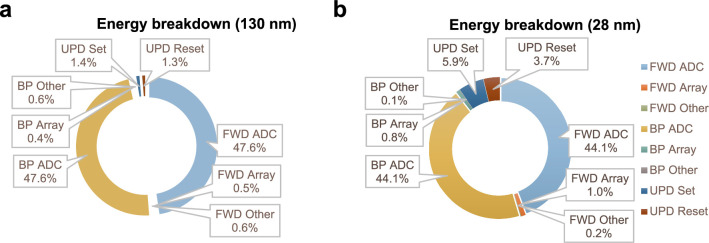
Energy breakdown of the memristor cores during one learning iteration. **a**, Energy breakdown of 130 nm. **b**, Energy breakdown of 28 nm. It shows the distribution of energy consumption across various components, with BP ADC and FWD ADC being the most significant at 95.2% and 88.2% for 130 nm and 28 nm technologies, respectively. The energy cost could be further minimized by refining the ADC design.

Moreover, we evaluated the energy consumption and latency of stochastic CIM computing system in this learning task (Supplementary Fig. 11 and Extended Data Table 1) and then compared it with a traditional CMOS-based graphics processing unit computing platform (Supplementary Note 9). The stochastic CIM computing system achieved a remarkable 44% boost in speed and conserved 153 times more energy. This speed could be further improved by employing high-parallel modulation methods, and the energy cost could be further minimized by refining the ADC design (Extended Data Fig. 9). Due to in-memory VMM and in situ sampling facilitated by the intrinsic physical randomness of reading and conductance modulation, memristor crossbars are capable of enabling both in situ learning and prediction, thus overcoming von Neumann bottleneck challenges.

## Discussion

Our research introduces an in-memory DBAL framework and an in situ learning method, both capitalizing on the stochastic properties of memristor modulation. This unique approach has been proven feasible and effective, as demonstrated in a robot skill learning task implemented on a neuro-inspired stochastic CIM system. These results highlight the potential superiority of memristor technology over current CMOS implementations, particularly in terms of efficiency and speed in robotic applications.

However, our research also presents opportunities for further exploration and improvement. For instance, the integration of specific computing circuits or general-purpose processing cores directly with memristor arrays could potentially replace the operations currently handled by external computers. Such a fully integrated chip could enable more efficient and sophisticated stochastic computations, pushing the boundaries of what is currently achievable in the field of robotics and AI.

It is important to note that, although our study highlights memristor-based DBAL’s potential in robotics, its effectiveness may differ across applications. Future research should validate our findings in broader tasks and real-world settings. Furthermore, as performance is tied to memristor properties, strategies for mitigating variations from manufacturing and operating conditions should be explored in future work.

## Methods

### In situ sampling via reading memristors

By adjusting the current state of the memristor, the dispersion of the read noise distribution can be modified, thereby tuning the read noise (Supplementary Note 1). This tuning of read noise enables the generation of sample values from various probability distributions. To generate a Gaussian probability distribution, the sum of the stochastic currents of three memristor cells can be used as a stochastic weight. It based on the Lindeberg–Feller central limit theorem, a more general form of the central limit theorem. The theorem states that a sum of random variables will tend toward a normal distribution, given that each variable’s influence tends toward zero. In our case, the three memristors are independent, and each has a limited influence, satisfying the conditions of the Lindeberg–Feller theorem (Supplementary Note 2), that is, the device-to-device variability and cycle-to-cycle variability in a memristor array can be regarded as a physical random variable (Supplementary Note 3). This can enable the sampling of random Gaussian weights across the entire memristor array in parallel.

### Measurements of the conductance transition

To analyze the conductance transition probability of the memristor, we performed set and reset tests with single voltage pulses on a 4K array. To obtain more continuous initial conductance state data, the target conductance state for writing was set to 20 values at uniform intervals between 0.7 μA and 3.7 μA at *V*_read_ = 0.2 V. Before testing, we regulated the same target conductance state for the entire array of memristors by writing verification method. Each device was then observed for conductance changes by applying five consecutive single voltage pulses of set or reset to exclude state stuck devices that accounted for a small percentage. At the time of testing, by using the write verification method again, we modulated the initial conductance state of the remaining devices to within ±0.3 μA at 0.2 V of the target conductance state and then applied a single set or reset pulse. In measuring the transfer characteristics under set operation, the operating voltage conditions of bit line (BL) is *V*_BL_ = *V*_set_ = 2.0 V, SL is *V*_SL_ = 0.0 V and word line (WL) is *V*_WL_ = 1.25 V. For reset, the operating voltage conditions were *V*_BL_ = 0.0 V, *V*_SL_ = *V*_reset_ = 1.95 V and *V*_WL_ = 2.55 V. The pulse width of set and reset is 100 ns and 50 ns, respectively. Finally, the conductance state is measured at read voltage *V*_read_ = 0.2 V as the subsequent conductance state.

### Stochastic models of memristors

A read noise model of memristors is developed using a double exponential distribution. Since the read noise *I*_noise_ is defined as the magnitude of the difference from the mean value of each device, *I*_noise_ follows a double exponential distribution with an average value of zero, that is, ${I}_{{{\mathrm{noise}}}} \approx {{\mathrm{Laplace}}}(0,{b}_{{{I}}})$. The parameter *b*_*I*_ indicates the dispersion of the read noise distribution, which can be determined by the current state *I* (Extended Data Fig. 2), and the read current *I*_read_ of the device is the current state *I* plus the read noise *I*_noise_, that is, *I*_read_ = *I* + *I*_noise_. Memristor Gaussian weights *w*_*I*_ in a BDNN can be simulated employing read currents accumulated from *N* = 3 devices

$${w}_{I} \sim \frac{1}{{V}_{{{\mathrm{read}}}}}\times \mathop{\sum }\limits_{n=1}^{N}{I}_{{{\mathrm{read}}},n}.$$

The conductance modulation model is based on our measured statistical distribution of the conductance transition. The models describe the average subsequent conductance values *μ*_set_ and *μ*_reset_ and the standard deviations *σ*_set_ and *σ*_reset_ at the current conductance state after applying a single voltage pulse of set or reset. The modulated conductance states obey a Gaussian distribution parameterized by

$$p({I}_{{{\mathrm{set}}}})={\rm{N}}{\rm{ormal}}\left({\mu }_{{{\mathrm{set}}}},{\sigma }_{{{\mathrm{set}}}}^{2}\right)$$

$$p({I}_{{{\mathrm{re}}}{{\mathrm{set}}}})={\rm{N}}{\rm{ormal}}\left(\;{\mu }_{{{\mathrm{re}}}{{\mathrm{set}}}},{\sigma }_{{{\mathrm{reset}}}}^{2}\right).$$

### Uncertainty quantification

There are different ways to quantify uncertainty depending on the needs of the active learning task. In this Article, the straddle algorithm^37^ is used to compute the prediction uncertainty for the robot’s skill learning task. The straddle algorithm in active learning prioritizes the selection of unlabeled data points based on their proximity to the decision boundary and the level of uncertainty associated with them. It does not distinguish between aleatoric and epistemic uncertainty. Instead, it assigns high scores to data points that are not only close to the decision boundary but also situated at a distance from previously sampled examples, aiming to explore regions of the feature space that are informative yet underrepresented in the current training set. To quantify the prediction uncertainty of a particular input data, the memristor BDNN needs to make multiple predictions on the input data to obtain the distribution of the prediction results. We repeat the forward propagation with stochasticity for *M* times, each time with a new sampling of the read current ${I}_{{{\mathrm{read}}},n}$. This results in *M* different outputs *y*_1_, *y*_2_, …, *y*_*M*_, which form a distribution of the network output *y*. With the distribution of the network output *y*, we calculate the mean *μ*_*y*_ and standard deviation *σ*_*y*_ of the prediction results to obtain the uncertainty *U*_*y*_ of the input data according to the straddle algorithm

$${U}_{y}=-|{\mu }_{y}|+1.96{\sigma }_{y}.$$

A negative value of *U*_*y*_ suggests that the model estimates a low probability (less than 5%) that the point lies on the decision boundary, indicating that the data point may be less informative for improving the model.

### mSGLD training method

In the proposed mSGLD training method, our loss function is set to

$${\mathrm{Loss}}=\beta \times {\rm{KL}}[q({w}_{I})||P(w)]+{{\mathbb{E}}}_{q({w}_{I})}[\log P(y|x,{w}_{I})],$$

where *q*(*w*_*I*_) denotes the distribution of the neural network’s memristor weights, *P*(*w*) signifies the prior Gaussian distribution of weights and *P*(*y|x*, *w*_*I*_) is the predictive distribution of outputs given inputs *x* and current weights *w*_*I*_. KL[‧] refers to the Kullback–Leibler (KL) divergence. This is a measure of how one probability distribution diverges from a second, expected probability distribution. In our case, we are measuring the divergence between the approximate posterior weight distribution *q*(*w*_*I*_) and prior *P*(*w*) = *N*(*μ*, *σ*). It can be calculated during forward propagation by log(*σ*/*σ*(*w*_*I*_)) + [*σ*^2^(*w*_*I*_) + (*μ*(*w*_*I*_) − *μ*)^2^]/*σ*^2^/2 − 0.5. ${{\mathbb{E}}}_{q(x)}[\cdot ]$ represents the expected log-likelihood term. It measures how well our model predicts the observed data. We can simply calculate the average log-likelihood of the output results of the network to approximate the expected value. *β* is the weight that balances the two loss terms. The loss function in our work is more complex than a traditional neural network owing to its Bayesian nature, and this complexity extends to the backpropagation equations. However, the core principles remain the same: we are still using gradient descent to optimize our weights, but the gradients are calculated with respect to a more complex loss function. The backpropagation process involves calculating the gradients of these two components of the loss function with respect to the memristor conductance of the network $\frac{\partial {{\mathrm{Loss}}}}{\partial I}$. Due to the physical constraints of our ESCIM system, this computation is performed on a digital computer. While the calculations are indeed more complex due to the Bayesian nature of the loss function, they can be efficiently computed using automatic differentiation tools available in modern machine learning libraries (Extended Data Fig. 8a), such as the popular PyTorch tool used in our work.

For smooth transition into the latter phase of mSGLD (Extended Data Fig. 8b), we choose a progressively smaller weight update percentage *p* to update only the key weights during iterative training. First, we sort the weights according to the absolute value of the conductance $\frac{\partial {{\mathrm{Loss}}}}{\partial I}$ from largest to smallest and then mark the top-ranked weights based on the current weight update percentage *p*. We repeat the gradient calculation process several times to select weight whose marking times exceed the set marking threshold. Then, one of the three devices is randomly selected and conductance modulation is performed according to equation (2). In the next iteration, the weight update ratio *p* becomes gradually smaller.

### Robot’s pouring skill learning task and robot simulator

The robot’s pouring skill learning task^5^ involves pouring liquid particles from a cup to a bowl. The prerequisite condition of this pouring task is that the robot must be holding a cup filled with liquid particles. The effectiveness of pouring action is determined by how much of the bowl is filled. The total number of particles successfully transferred into the bowl is estimated by counting the particles that remain within the bowl’s 3D axis-aligned bounding box. This method provides a quantifiable measure of learning performance of BDNN model.

We used a 3D tabletop environment simulator with a robot using a digital computer^5^. A dynamic 3D space was created by using the PyBullet physics engine, where the robot can perform pouring tasks. Our experiments involved objects derived from a real-world dataset of bowls and cups. We introduced randomness to the simulation by independently scaling the diameter and height of the bowls and cups. The objects’ physical properties, such as mass and friction, were assigned on the basis of a truncated Gaussian distribution, ensuring a high degree of variability in the simulation. In addition, the characteristics of the liquid particles, including number, radius and density, were randomly sampled, guaranteeing that each trial in training or testing was unique. For the simulation and planning phases, PyBullet was employed for tasks such as forward kinematics, collision detection and visualization. The robot’s seven-degree-of-freedom arm was controlled with IKFast handling inverse kinematics and RRT-Connect used for planning free-space arm movements. For motions that required the robot’s gripper to follow a Cartesian path, we used randomized gradient descent as a constrained motion planner. The robot’s actions were performed through a sequence of planned arm or gripper configurations, managed by a position controller. A rigid attachment constraint was applied when the robot grasped an object, mirroring the real-world scenario where the object remains stable relative to the robot’s hand during movement. This comprehensive simulation framework allowed us to thoroughly test and refine our proposed methods in a controlled yet realistic and complex 3D environment.

### Experiment system setup

The ESCIM system integrates three 4K memristor chips, facilitating in situ VMM through a crossbar structure. The developed 4K memristor chip with a 1T1R structure integrated 32 × 128 memristors. This ESCIM system includes a core board equipped with a field-programmable gate array for signal control and communication, a transimpedance amplifier and an ADC board for current quantization, a digital-to-analog converter board for voltage supply, a mother board for signal conversion and a device-under-test board connected to three socket boards that can hold a 4K memristor chip each. Detailed specifications of the ESCIM system can be found in our early work^19^. Via a network port, the ESCIM system is connected to a digital computer that runs the 3D tabletop environment simulator with a robot.

The size of the memristor BDNN implemented by the ESCIM system is 11 × 50 × 50 × 2. This network is structured to incorporate 11 input neurons, which encode the context parameters and control parameters. The output layer of the network consists of two output neurons representing successes and failures of actions. The selection of 50 nodes for the two hidden layers was based on empirical testing (refer to Supplementary Note 6). In the predict process of memristor BDNN, the current of memristor cells are measured in a row-by-row manner. This process is meticulously controlled via a field-programmable gate array, ensuring high voltage across the WL to activate transistors fully, while a reading voltage *V*_read_ = 0.2 V is simultaneously applied between the SL and the BL for each row. The resultant read currents from the cells are digitized using 64 channels of ADCs simultaneously. These digital values are then used to generate the network’s predictions, processed through a digital computer. In the in situ learning process of memristor BDNN, the digital computer sends the address and modulation direction of cells selected by mSGLD to the ESCIM system. The KL value of the last layer of the network was calculated in the demonstration, and then the selected cells were modulated one by one using the operation condition as described in ‘Measurements of the conductance transition’ section in Methods.

## Supplementary information

Supplementary Information Supplementary Figs. 1–11, Tables 1–3 and Notes 1–9, Caption for Supplementary Video 1 and References.

Supplementary Video The video shows the visualization results for three different cases (bowl and cup pairs), including small green bowl and yellow cup, yellow bowl and red cup, and blue bowl and large orange cup. The visualization results for different amounts of newly added highest-uncertainty data show that BDNN’s classification performance is enhanced by highest-uncertainty data. The visualizations of the pouring results show the robot pouring the beads from the cup into the bowl.

## Source data

Source Data Fig. 2 Statistical source data for Fig. 2a–e.

Source Data Fig. 4 Statistical source data for Fig. 4b–e.

Source Data Extended Data Fig. 2 Statistical source data for Extended Data Fig. 2.

Source Data Extended Data Fig. 3 Statistical source data for Extended Data Fig. 3.

Source Data Extended Data Fig. 4 Statistical source data for Extended Data Fig. 4.

Source Data Extended Data Fig. 5 Statistical source data for Extended Data Fig. 5.

Source Data Extended Data Fig. 6 Statistical source data for Extended Data Fig. 6.

## Data Availability

The dataset used in this study is publicly available via Zenodo at 10.5281/zenodo.14168532 (ref. ^38^). Source data are provided with this paper.

## Extended data

**Extended Data Table 1:** Energy cost and latency breakdown of the memristor-based ESCIM in performing a learning iteration

Phases	Process	130 nm		28 nm	
		Energy (nJ)	Latency (μs)	Energy (nJ)	Latency (μs)
FWD		104.46	0.48	18.11	0.26
BP		104.00	0.45	18.00	0.25
UPD	SET	2.93	2.55	1.76	2.04
	RESET	2.78	2.55	1.12	2.04
**Summary**		**214.17**	**6.04**	**38.98**	**4.59**

## Code availability

The code that needs to run on the ESCIM hardware platform is available via GitHub at https://github.com/YudengLin/Code_ESCIM_Hardware and Zenodo at 10.5281/zenodo.14168528 (ref. ^39^). Other codes are available via GitHub at https://github.com/YudengLin/BDAL_for_Robot_Skill_Learning and Zenodo 10.5281/zenodo.14168532 (ref. ^38^). The 3D tabletop environment simulator with a robot refers to the code on GitHub at https://github.com/caelan/LTAMP. The memristor and BDNN model refers the code on GitHub at https://github.com/YudengLin/memristorBDNN.
