# Supplementary material for: Deep Bayesian active learning using in-memory computing hardware
Source: Nat Comput Sci. 2024 Dec 23;5(1):27–36. doi: 10.1038/s43588-024-00744-y (PMC11774754; doi:10.1038/s43588-024-00744-y)
Supplement: Supplementary file 1 — Supplementary Figs. 1–11, Tables 1–3 and Notes 1–9, Caption for Supplementary Video 1 and References. [file 43588_2024_744_MOESM1_ESM.pdf]

nature computational science

Supplementary information

https://doi.org/10.1038/s43588-024-00744-y

Deep Bayesian active learning using in-memory computing hardware

In the format provided by the authors and unedited

This Supplementary Information contains:

Supplementary Figures 1 – 11 **3**

Supplementary Fig. 1 | Weight mapping procedure to deploy the BDNN on memristor crossbar arrays. 3

Supplementary Fig. 2 | Forward propagation to predict using memristor BDNN with stochastic read currents. 4

Supplementary Fig. 3 | Uncertainty calculation procedure. 5

Supplementary Fig. 4 | mSGLD training procedure to update the memristor BDNN model with training data. 6

Supplementary Fig. 5 | Reduce weight update percentage scheduler with various decay factor γ in our BDAL simulation experiment based on MNIST classification task. 7

Supplementary Fig. 6 | Reduce weight update percentage scheduler with various initial p in our BDAL simulation experiment based on MNIST classification task. 8

Supplementary Fig. 7 | Comparison of accuracy on test data set between proposed mSGLD and regular stochastic gradient descent (SGD) across 100 queries of BDAL simulation experiment based on MNIST classification task. 9

Supplementary Fig. 8 | Accuracy on test data set comparison between SGLD with floating-point gradient and mSGLD with binarizing gradient across 100. 10

Supplementary Fig. 9 | Pseudocode of deep Bayesian active learning framework in our robot's skill learning task. 11

Supplementary Fig. 10 | Test accuracy of BDNN in robot skill learning task over a retention time at 125°C. 12

Supplementary Fig. 11 | Circuit modules of the simulated memristor core to evaluate the speed and energy cost of the FWD phase and the BP phase. 13

Supplementary Tables 1 – 3 **14**

Supplementary Table 1 | Architecture of the BDNN used for the robot skill learning task. 14

Supplementary Table 2 | NVIDIA Tesla A100 performance metrics. 15

Supplementary Table 3 | Energy cost and latency breakdown of the GPU in performing a learning iteration. 16

Supplementary Notes 1 – 9 17

Supplementary Note 1: Characteristics of read noise 17

Supplementary Note 2: Readout currents from three memristors represent a normally distributed weight 18

Supplementary Note 3: Impact of device-to-device variability and read-to-read variability on constructing a Gaussian weight 20

Supplementary Note 4: Pseudocodes for weight mapping, network inference, network training, uncertainty estimating and active learning framework 22

Supplementary Note 5: BDAL simulation experiment based on MNIST handwritten digit image classification task 24

Supplementary Note 6: Choosing the topology of BDNN 27

Supplementary Note 7: Technical information about the learning process of robot's skill learning task 28

Supplementary Note 8: Impact of read-to-read variability on network performance over time 29

Supplementary Note 9: Energy consumption and latency estimation 31

Caption for supplementary video 1 36

References 37

Supplementary Figures

```
Procedure weightMapping
    input: TargetConductance
    Output: FinalConductance
    # Loop until the conductance is within the acceptable range
    while TotalPulse<=MaxPulse do
        # Measure the current conductance value
        CurrentConductance = measureConductance(memristor)
        # Check if the conductance is within the error margin of the target
        if abs(CurrentConductance-TargetConductance)<=ErrorMargin (0.3 μA) then
            FinalConductance = CurrentConductance
            return FinalConductance # Exit the loop as the conductance is within
            the error margin of the target
        else
            programPulse(memristor) # If not, apply another program pulse
        end if
    end while
End Procedure
```

Supplementary Fig. 1 | Weight mapping procedure to deploy the BDNN on memristor crossbar arrays. This conductance mapping process is executed on the our ESCIM hardware platform.

Procedure forwardPropagation

input: data *x*, weight prior *p(w)*

output: prediction *y*, Kullback-Leibler divergence L_{KL}

```
# Read cell currents process
for each row in memristor arrays
    # Set WL voltage to high to turn on transistors
    V_WL=High
    # Apply read voltage V_read=0.2 V between SL and BL
    V_BL-SL=V_read
    # Convert SL currents to digital values I_read using ADCs
    I_read = ADC(V_read × G).
end for
```

# Make a prediction for data x

Layer input x_{input} = data *x* *# Prepare input for the first layer*

$L_{KL} = 0$

for each layer in memristor BDNN do

$x_q = quantize(x_{input},\ 8)$; *# Quantize layer input to 8-bits*

$w_I \sim \frac{1}{V_{read}} \times \sum_{n=1}^{3} I_{read,n}$; *# Calculate Gaussian weight*

$O_l = activationFunction\left(x_q \cdot w_I\right)$ *#Calculate layer's output*

L_{KL} += KL(q(w_I), *p(w)*)

if is last layer then

return Prediction $y = O_l$ and L_{KL}. *# Exit the loop*

else

$x_{input} = O_l$ *# Set the current layer's output as the next layer's input*

end if

end for

End Procedure

Supplementary Fig. 2 | Forward propagation to predict using memristor BDNN with stochastic read currents. The yellow square represents that this reading cell conductance process is executed on the our ESCIM hardware platform. The rest of the part is using a digital computer to implement.

Procedure calculateUncertainty
Input: Data *x*, Forward propagation times *M=5*
Output: Uncertainty U_y

# Perform forward propagation M with stochasticity
for i from 1 to M do
# Forward propagation: compute the predictions
Prediction *y* = **forwardPropagation**(data *x*)
# Store the prediction in the array
NetworkPredictions array append *y*
end for

# Calculate the mean of the outputs
μ_y = mean(NetworkPredictions)
# Calculate the standard deviation of the outputs
σ_y = standardDeviation(NetworkPredictions, μ_y)
# Calculate the uncertainty of the prediction
U_y = -abs(μ_y) + 1.96 * σ_y
return U_y
End Procedure

Supplementary Fig. 3 | Uncertainty calculation procedure. The green square indicates that this part involves the ESCIM hardware platform as defined above. The rest of the part is using a digital computer to implement.

```
Procedure mSGLDTraining
    Input:TrainingData x, UpdatePercentage p
    Output: TrainedNeuralNetwork

    # Loop over the number of epochs
    for epoch in range(1, Epochs+1) do
        # Forward propagation: compute the predictions
        Predictions y = forwardPropagation(data x)
        # Compute the loss
            Loss = β · KL[ q(w_I) ‖ P(w)] + E_{q(w_I)}[log P(y|x, w_I)]
        # Backward propagation: compute the gradient of the loss
        Gradient = backwardPropagation(Loss)
        # Mark top p% of the max absolute value of the weights' gradients
        KeyWeights = markTopPWeights(Gradient, p)
        # Repeat gradient calculation and marking process
        for repeat in range(1, MarkingThreshold+1) do
          Gradient = recalculateGradient(data x)
          updateMarkingCount(KeyWeights)
        end for

        # Update only the weights whose marking times exceed the threshold
        for weight in KeyWeights do
          if weight.MarkingCount > MarkingThreshold then
            # Random select one device
            Device = randomSelectDevice(weight, 1)
            # Modulate the selected device conductance
            if Device.Gradient > 0 then
                setDevice(V_bl=2.0, V_sl=0.0, V_wl=1.25)
            else if Device.Gradient < 0 then
                resetDevice(V_bl=0.0, V_sl=0, V_wl=2.55)
        # Reduce the weight update percentage
        p = reduceUpdatePercentageScheduler (p, epoch)
      end for

    end for
End Procedure
```

Supplementary Fig. 4 | mSGLD training procedure to update the memristor BDNN model with training data. The yellow square represents that this modulating part is executed on the ESCIM hardware platform. The green square indicates that this part involves the ESCIM hardware platform as defined above. The rest of the part is using a digital computer to implement.

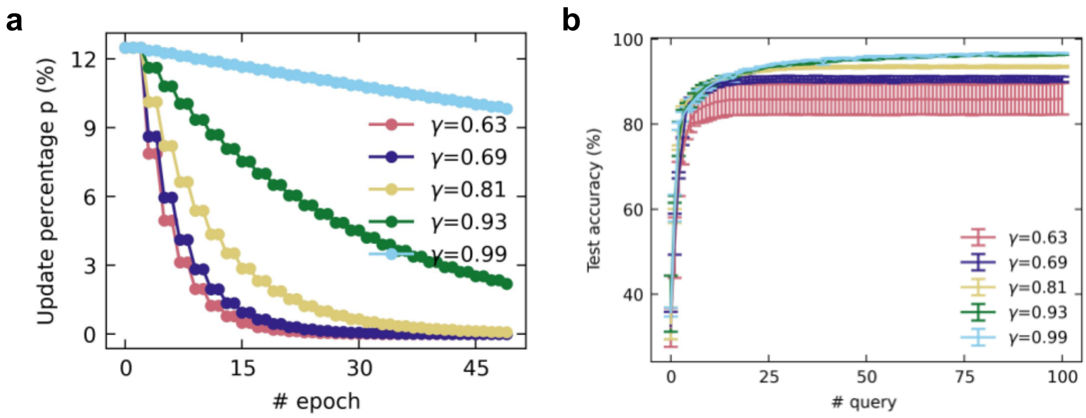

Supplementary Fig. 5 | Reduce weight update percentage scheduler with various decay factor γ in our BDAL simulation experiment based on MNIST classification task. a, decline in update percentage *p* over training epochs for various values of decay factor γ. **b,** test accuracy as a function of the number of queries for various values of decay factor γ. The error bar is the standard deviation (SD) of three repeated experiments. Data are presented as mean values +/- SD.

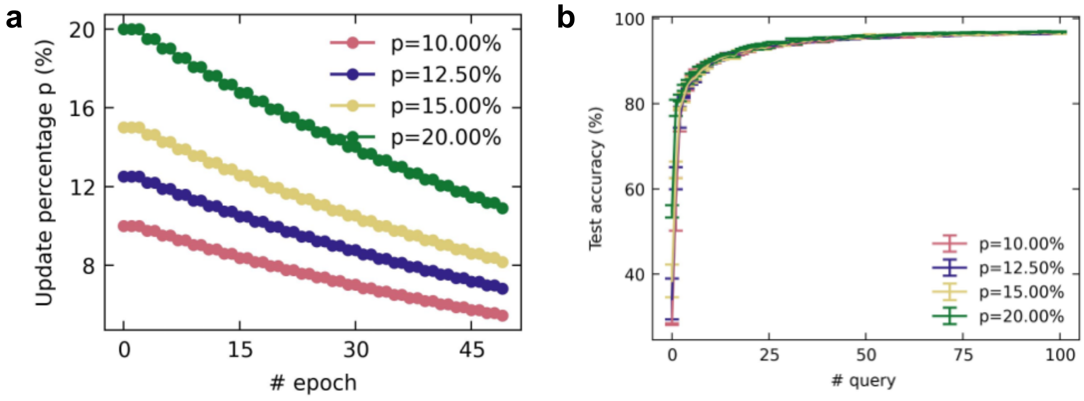

Supplementary Fig. 6 | Reduce weight update percentage scheduler with various initial *p* in our BDAL simulation experiment based on MNIST classification task. a, Weight update percentage decreasing over the number of epochs for different initial *p*. **b,** Test accuracy as a function of the number of queries for various values of initial *p*. The error bar is the SD of three repeated experiments. Data are presented as mean values +/- SD.

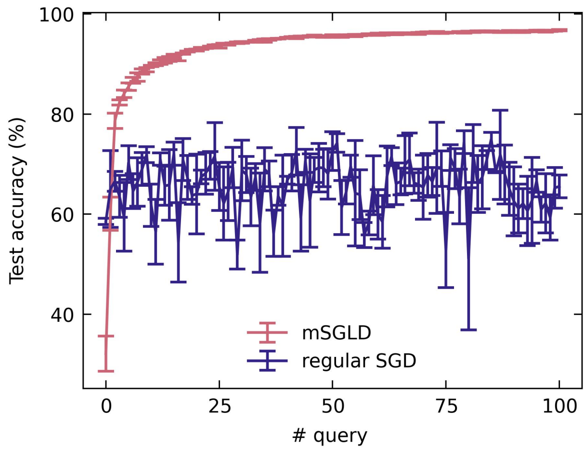

Supplementary Fig. 7 | Comparison of accuracy on test data set between proposed mSGLD and regular stochastic gradient descent (SGD) across 100 queries of BDAL simulation experiment based on MNIST classification task. The error bar is the SD of three repeated experiments. Data are presented as mean values +/- SD.

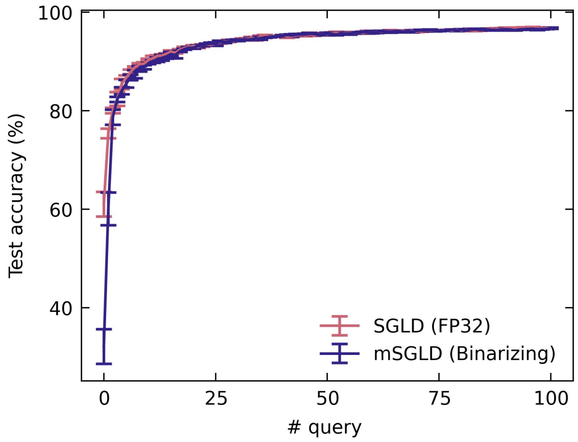

Supplementary Fig. 8 | Accuracy on test data set comparison between SGLD with floating-point gradient and mSGLD with binarizing gradient across 100 queries. It demonstrates similar performance trends with slight variations in accuracy of our BDAL simulation experiment based on MNIST classification task. The error bar is the standard deviation of three repeated experiments.

```
Procedure mainDeepBaysainActiveLearning
    Input: InitialTrainingData

    # Step 1: Train the initial BDNN model ex-situ
    InitialBDNN = exsituTraining(InitialTrainingData)
    # Step 2: Deploy the model on memristor crossbar arrays
    mBDNN = weightMapping(InitialBDNN.TargetConductance)
    # Initialize the training dataset with the initial data
    TrainingDataset = InitialTrainingData

    # Step 3:Deep Bayesian active learning loop
    while Quota > 0 do
        # Step 3a: calculate uncertainty for the unlabelled dataset
        PredictionUncertainties = calculateUncertainty(UnlabelledDataset)
        # Step 3b: Select the data sample with the highest uncertainty
        SampleToQuery= selectHighestUncertainty(PredictionUncertainties)
        # Step 3c: Query for the label of the selected sample
        QueriedLabel = QueryLabel.planExecute(SampleToQuery)
        Quota -= 1
        # Step 3d: Add the newly labeled sample to the training dataset
        TrainingDataset add (SampleToQuery, QueriedLabel)
        # Step 3e: Perform in situ learning with the updated training dataset
        mBDNN = mSGLDTraining (TrainingDataset)
        # Check if performance meets expectations or continue if quota allows
        if performanceMeetsExpectations(mBDNN) then
            break
        end if
    end while
End Procedure
```

Supplementary Fig. 9 | Pseudocode of deep Bayesian active learning framework in our robot's skill learning task. The green square indicates that this part involves the ESCIM hardware platform as defined above. The rest of the part is using a digital computer to implement.

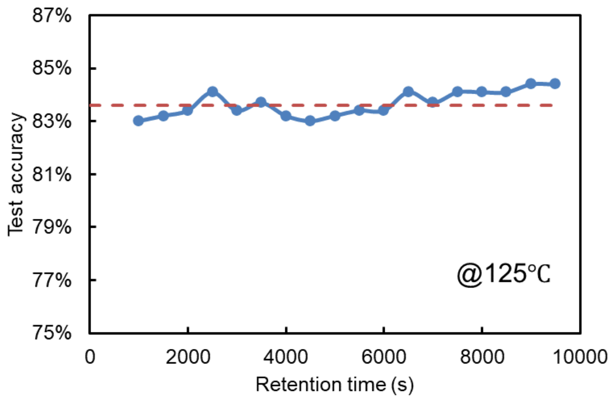

Supplementary Fig. 10 | Test accuracy of BDNN in robot skill learning task over a retention time at 125°C. This demonstrates a relatively stable performance with accuracy levels mostly hovering around the 83% mark.

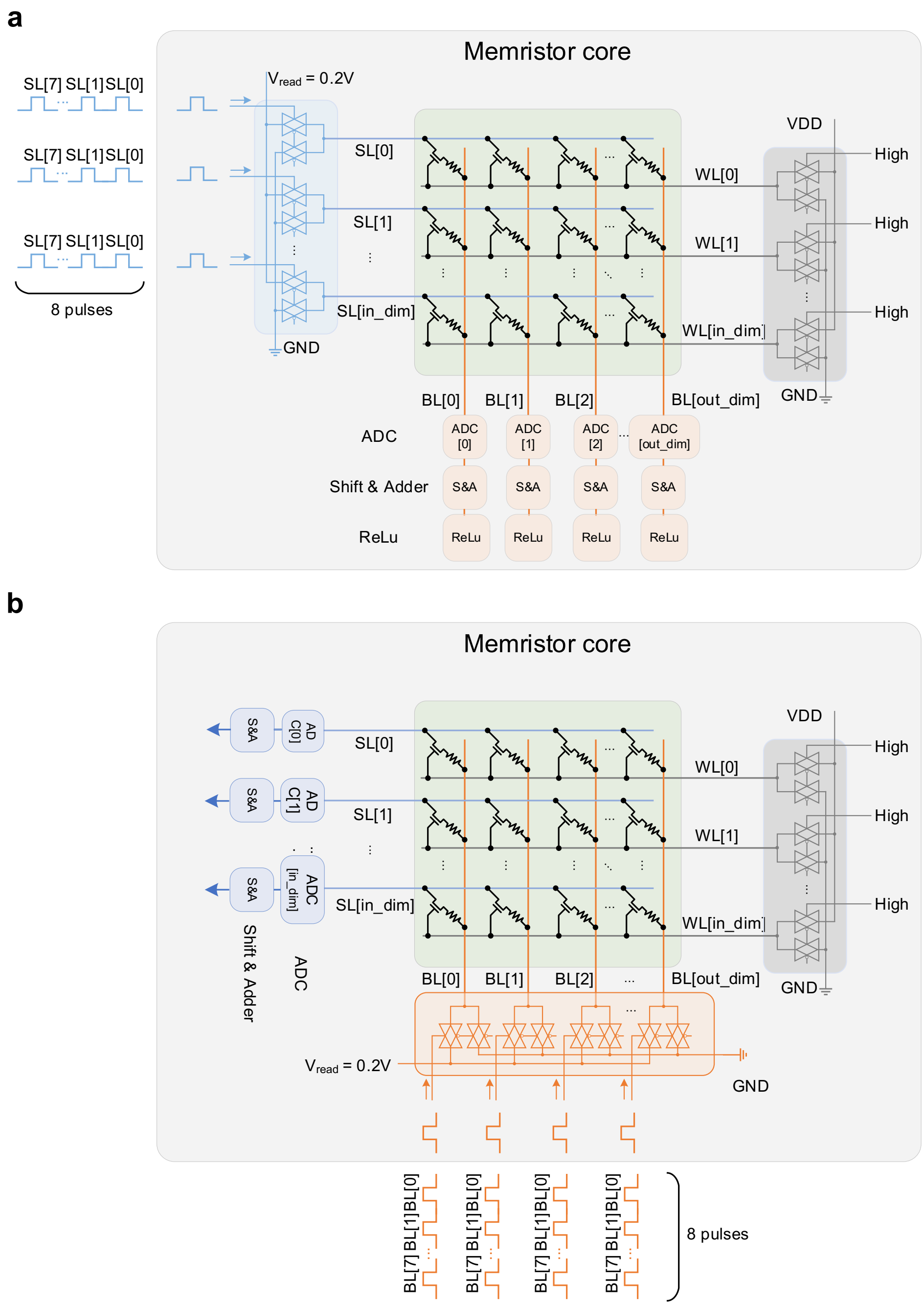

Supplementary Fig. 11 | Circuit modules of the simulated memristor core to evaluate the speed and energy cost of (a) the FWD phase and (b) the BP phase.

Supplementary Tables

Supplementary Table 1 | Architecture of the BDNN used for the robot skill learning task.

**Weight Layer**	**Input neurons of weight layer (n_{in_dim})***	**Output neurons of weight layer (n_{out_dim})**	**No. of random weights (or samples/multiplications/additions per forward pass) (n_{weight})**
w_1	12	50	600
w_2	51	50	2550
w_3	51	2	101

* With bias input

Supplementary Table 2 | NVIDIA Tesla A100 performance metrics.

Features	Values
TDP [1]	P_{GPU}=400 W
Peak OPS (32-bit floating-point number computation) [1]	OPS_{GPU}=19.5 TOPS
FP32 cores [1]	N_{core}=6912
High bandwidth memory (HBM) latency [2]	T_{GPU}=404 ns
Random number generation throughput [3] (normal distribution, 32-bit floating-point number)	S_{GPU}=236.6 GSamples/sec

Supplementary Table 3 | Energy cost and latency breakdown of the GPU in performing a learning iteration.

Phases		Energy (μJ)	Latency (μs)
FWD	Sample	1.65	2.44
	VMM	0.27	
BP	VMM	0.22	0.81
UPD	Sample	3.30	4.88
	VMM	0.53	
**Summary**		**5.97**	**8.13**

Supplementary Notes

Supplementary Note 1: Characteristics of read noise

In our study, the read noise of a memristor is quantified as the difference between the observed read current and the average read current of the device, under the same current state. This means that for each measurement of the read current, the read noise is calculated by subtracting the average read current (calculated over multiple readings) from each individual reading. This approach helps in identifying the variability or fluctuation in the read current that can occur due to inherent device characteristics.

The distribution of these read noise values, across different reading cycles and different memristor devices at a selected target current state, has been modelled using a Laplace distribution. The Laplace distribution is particularly suited for this purpose due to its ability to effectively model data that exhibit a sharp peak at the mean and heavier tails, which is typical in RTN noise data where most values cluster around the mean but with some significant deviations due to outliers or sporadic spikes in read current. The Laplace noise distribution encompasses the cycle-to-cycle and device-to-device variations.

We have plotted the probability density functions of the read noise for various memristor conductance states and fitted these distributions with the Laplace model to better understand the noise characteristics in relation to the conductance of the device (Extended Data Fig. 2). These plots provide a clear visual representation of how the read noise distribution changes with memristor conductance.

We also have calculated the values of ΔI/I following the definition in the reference article [4, 5] in our previous work [6]. To obtain statistical data, we selected 325 devices in the current window ranges from 2×10^{-7} to 2×10^{-6} A at V_{read} = 0.1 V, whose read current fluctuations show RTN behaviors. The result is shown in the previous work's supplementary material. When compared the data from the literature [4, 5] over current window ranges, we can find that the trend of relationship and the order of magnitude of ΔI/I are aligned with each other.

For the monotonical trend of ΔI/I vs I, since the sum computation of VMM is realized based on the accumulation of the value of the current, we defined the read noise as the absolute variation of current value, instead of using the relative value ΔI/I. Extended Data Fig. 3 in our paper presented relationship between the scale factor *b* of the absolute read noise and current state. It shows that the overall dispersion of absolute read noise is higher in the middle- conductance states than that in the low- and high-conductance states.

Supplementary Note 2: Readout currents from three memristors represent a normally distributed weight

According to the classical central limit theorem, the sum of multiple independent and identically distributed random variables conforms to a normal distribution. However, the Lindeberg-Feller central limit theorem, a more general form of the central limit theorem, states that a sum of random variables will tend towards a normal distribution, given that each variable's influence tends towards zero. This means that the variables do not necessarily need to have the same distribution. In our case, the three memristors are independent, and each has a limited influence, satisfying the conditions of the Lindeberg-Feller theorem. Furthermore, the memristors we used follow a Laplace distribution, which is similar in shape to a normal distribution. Therefore, the sum of the output currents from these three memristors can approximate a normal distribution to a certain extent. Furthermore, we have conducted additional experiments to validate our approach. Extended Data Fig. 4 shows the distribution of the weights sampled over different read cycles. This confirms the similarity of these distributions to a normal distribution, validating our use of three memristors to represent a normally distributed weight.

Further support for our model come in our prior work [7], whose Fig.9 shows that when the number of memristors (N) used to represent a Gaussian weight exceeds two, the distribution of the total current from these memristors can be well-fitted with a Gaussian distribution. This suggests that increasing the number of memristors helps in achieving a distribution that closely mimics an ideal Gaussian distribution, which is crucial for accurately modelling Gaussian weights in BDNN.

The decision to set N=3 stems from a balance between simulation accuracy and hardware costs. While increasing the number of memristors could reduce the KL divergence and enhance output accuracy (Fig.13 and Fig. 14 in our prior work [7]), it would also raise the complexity and cost of the hardware. The experimental data suggests that three memristors per weight strike a good balance, adequately fitting the Gaussian distribution while keeping the KL divergence relatively low. This number represents a practical compromise that ensures quality output while managing system complexity and implementation costs.

Regarding the independence of mean and variance in the normally distributed weights of the BDNN, we acknowledge that we cannot independently adjust the mean and variance of the normal distribution by tuning the current magnitudes of the three memristors alone. However, we have adopted a hardware-software co-optimization method [6] that takes into account the constraints of the memristor's mean and the scale factor b during the training process to achieve appropriate Gaussian weights.

Specifically, in the ex-situ learning phase, we utilize the relationship between the scale factor b and the current I. We backpropagate the objective function error

caused by the scale factor b to calculate the gradient of I. Then, we update I directly based on this gradient. This update process continues until the training algorithm finds the optimized conductance, where b is also optimized. Therefore, the memristor variational inference directly obtains the appropriate current state of each cell. This approach allows us to achieve Gaussian weights that meet the constraints of the memristor's mean and variance.

Supplementary Note 3: Impact of device-to-device variability and read-to-read variability on constructing a Gaussian weight

Read-to-read variations primarily stem from randomness during operation, such as the randomness of physical particles motion. It represents the noise or fluctuations in the readout of a single memristor's conductance during repeated measurements under similar conditions. Therefore, read-to-read variations can potentially lead to weight deviations over time. This form of variability is evident in the spread of each individual histogram in Extended Data Fig. 5a, where the read current of a single device is plotted. On the other hand, device-to-device variations primarily originate from microscopic differences during the manufacturing process of memristors, such as differences in the microscopic structure of materials and minor errors during manufacturing. This variability manifests as the distinct shapes and spreads of the read current distributions for each device, as shown in Extended Data Fig. 5a. The device-to-device variability results in different readout conductance distributions for each memristor, even if they are programmed to the same conductance state. However, these variations are determined once the memristor device is manufactured, and therefore, they do not change over time. As a result, device-to-device variations do not lead to weight deviations over time.

Although the distributions for individual devices do not perfectly conform to a Gaussian shape—likely reflecting both read-to-read variability and device-to-device variability—their differences are critical to understanding the overall behaviour when these devices are combined. The histograms in Extended Data Fig. 5b&c demonstrate how these two types of variability are integrated. When we sum the read currents of three devices at a specific current level, as shown in Extended Data Fig. 5b for combinations [1,2,3], [4,5,6], and [7,8,9], the resulting distributions closely approximate Gaussian curves, as indicated by the fitted models. This is a clear indication that the sum of independent random variables, which in this case are the read currents from different memristor devices, tends towards a Gaussian distribution, a phenomenon that is well-aligned with the principles of the Central Limit Theorem.

Similarly, in Extended Data Fig. 5c, the sum current distributions for different combinations of devices [1,4,7], [2,5,8], and [3,6,9] at different current levels, show that despite each device's unique distribution due to device-to-device variability, the aggregated conductance values yield a Gaussian distribution. This is evidenced by the close match between the measured distributions and the Gaussian fits, underscoring the robustness of our approach in synthesizing Gaussian weights from the inherent variabilities observed in memristor devices.

In conclusion, as we combine device-to-device variability and read-to-read variability from multiple memristors and across numerous read cycles, the Central Limit Theorem comes into play. While each individual noise profile might be different distribution, the aggregate effect of combining several such distributions,

each with its own mean and variance, leads to a distribution that tends towards Gaussian. This is because the theorem assures that the sum of a sufficiently large number of independent random variables, regardless of their individual distributions, will approximate a normal distribution, provided no single variable dominates.

Supplementary Note 4: Pseudocodes for weight mapping, network inference, network training, uncertainty estimating and active learning framework

We have included five pseudo-codes for weight mapping, network inference, network training, uncertainty estimating and active learning framework in the revised supplementary materials (Supplementary Figs. 1-4, 9). We also explicitly highlight the operations carried out on our ESCIM hardware platform. These operations, which are marked with a yellow box in the pseudo-code, include mapping weights, reading cell conductance, and modulating conductance. These operations are integral to the functioning of our system and are executed directly on our ESCIM hardware system. This will provide a step-by-step guide, helping readers to better understand the process and the underlying principles of our method. Here is a summary of the pseudo-codes that we have provided in the supplementary materials:

1. **Procedure weightMapping** (Supplementary Fig. 1): This procedure maps the target conductance to the memristor crossbar arrays. It involves a loop that continues until the conductance is within an acceptable range. This is executed on the our ESCIM hardware platform.

2. **Procedure forwardPropagation** (Supplementary Fig. 2): This procedure describes the forward propagation process in the memristor BDNN. It involves reading cell currents and making predictions based on the data. The reading cell conductance process is executed on the our ESCIM hardware platform. The rest of the part is using a digital computer.

3. **Procedure calculateUncertainty** (Supplementary Fig. 3): This procedure calculates the uncertainty of the prediction. It involves performing forward propagation with stochasticity, calculating the mean and standard deviation of the outputs, and calculating the uncertainty of the prediction.

4. **Procedure mSGLDTraining** (Supplementary Fig. 4): This procedure describes the mSGLD training process. It involves looping over the number of epochs, forward propagation, computing the loss, backward propagation, marking key weights, updating weights, and reducing the weight update percentage. The modulating device condunctance is executed on the ESCIM hardware platform. For smooth transition between the two training phases of SGLD, we introduce a reducing the weight update percentage scheduler during training. The weights are updated by selecting a subset based on their gradient magnitudes and the percentage of weights updated (p) decreases progressively with each training iteration. The scheduler also helps in reducing the impact of conductance stochasticity by focusing updates on weights with significant gradients, thereby stabilizing the learning process as it converges.

5. **Procedure mainDeepBaysainActiveLearning** (Supplementary Fig. 9): This procedure describes the main active learning framework. It involves training the initial BDNN model ex-situ, deploying the model on memristor crossbar arrays, initializing the training dataset, and executing the active learning loop.

However, due to the experimental nature of our ESCIM system, some operations are currently implemented on a digital computer. Notably, the most time and energy-consuming operation implemented on the digital computer is the backpropagation

algorithm. Previous research [8] has shown that the backpropagation algorithm can also be efficiently implemented directly on memristor arrays, which is a promising direction for future development of our system.

In addition, the operations required to operate the neural network in forward, backward, and update modes, which are currently handled by the digital computer, can be designed and integrated into specialized peripheral circuits. These circuits are primarily based on digital logic and require a limited number of gates. Their functions are rather efficient in advanced silicon technology nodes, making them a viable and efficient solution for these operations.

Supplementary Note 5: BDAL simulation experiment based on MNIST handwritten digit image classification task

We conducted a BDAL simulation experiment based on MNIST handwritten digit image classification task to thoroughly discuss the management of noise in mSGLD, using regular stochastic gradient descent (SGD) instead of Langevin, and the impact of binarizing the gradient. The simulation experiment was set up with 100 initial training data points, with a uniform data distribution for each class. The BDNN's structure was 784×256×128×10, used to predict the input data of 0-9, a total of 10 digital categories with an input dimension of 784 (image size 28×28). The uncertainty calculation used the maximise mean standard deviation method [9]. A total of 100 queries were made, each time querying the labels of 50 samples with the largest uncertainty amplitude in unlabelled data pool, and the queried samples and labels were added to the training dataset. After obtaining the updated training dataset, the network trained for 50 epochs. The overall process of the experiment was consistent with the pseudocode added above.

In the end of active learning, the training dataset finally contained 5100 sample images, which is only about 10% of the original training dataset samples (the original training sample number is 60,000). The final classification accuracy baseline of the BDNN network simulated using floating-point computation was 96.71 %. In the discussion of the memristor BDNN network, the network weights were implemented using the memristor read and write noise model established in Method 3 in the manuscript. Under the appropriate weight update percentage decay scheduler, the final classification accuracy could reach a comparable 96.94%.

1) Management of noise in mSGLD

For the weight update percentage, a scheduler reducing the weight update percentage was utilized to diminish the update percentage of the memristor BDNN by a decay factor γ. Commencing from the initial update percentage p, the update percentage was multiplied by γ after every alternate epoch, perpetually adjusting the update rate throughout the mSGLD training process. In our empirical experiments, other schedulers of a similar exponential decay type exhibit approximately the same effect.

We first analyzed the influence of the decay rate. In the simulation experiment, we set a fixed initial p=12.5% and set 5 different decay factors of γ. Within 50 training epochs, the five change curves of the weight update percentage are depicted in Supplementary Fig. 5a, and the corresponding classification accuracy of various decay factors is illustrated in Supplementary Fig. 5b. As observable in Supplementary Fig. 5b, when the decay rate is comparatively swift, such as a diminutive γ=0.63 and 0.69, the ultimate performance of the network is significantly constrained. This is attributed to the excessively rapid weight update decay and the minuscule weight update ratio in the early training phase. mSGLD cannot effectively emulate a stochastic gradient algorithm, as it transitions to the Langevin dynamic MH process prematurely, impeding the network's capacity for effective learning. Consequently, the network's uncertain

learning capacity is compromised, and it fails to select the most informative samples during querying. Despite the increase in the number of queries, the performance of the network does not show significant improvement. However, when the decay speed of the weight update percentage decelerates, such as from γ=0.81 to a more suitable γ=0.93 and 0.99, mSGLD can gradually transition between the two phases more smoothly, thereby effectively enhancing the network's performance.

Subsequently, we analysed the impact of the initial weight update ratio *p*. In the simulation experiment, we established a fixed initial decay factor γ=0.975 and configured four different *p* values. During the 50-epoch training process, the four change curves of the weight update percentage are depicted in Supplementary Fig. 6a, and the corresponding classification accuracy on the test dataset is illustrated in Supplementary Fig. 6b. As observable in Supplementary Fig. 6b, a larger initial *p* value can enhance the network's classification performance in the initial stage. But following a substantial number of queries, the network's classification accuracy essentially remains constant. An excessively large weight update ratio will incur additional conductance modulation time and energy consumption. Therefore, the selection of the initial *p* value should consider the number of the task query time and the sensitivity of the hardware overhead.

2) Using regular SGD instead of mSGLD

For using regular stochastic gradient descent (SGD) instead of Langevin, extensive research has been conducted on implementing online learning of memristor hardware using SGD. Due to the existence of memristor modulation noise, partial weight updates are usually chosen, such as using regular SGD to solve the gradient and then selecting weights for update that have a gradient greater than a set gradient threshold. In our regular SGD simulation experiment, we set the gradient threshold to 1e-6. The regular SGD simulation results are shown in Supplementary Fig. 7. It can be seen that the final classification accuracy of regular SGD is only about 68%, compared to final classification accuracy mSGLD, which is 96.94%. It is worth noting that only 5100 samples were used here, which is only about 10% of the original training dataset samples (the original training sample number is 60,000). In regular SGD, due to the challenges of effective weight updates caused by memristor modulation noise and the inability of the regular SGD algorithm to learn uncertainty, it cannot select the most informative samples well at each query. Even with the increase in the number of queries, the performance of the network cannot be effectively improved.

3) Impact of binarizing the gradient

Regarding the impact of binarizing the gradient, we discussed the comparison between simulation floating-point gradient and binary gradient, highlighting the advantages of binary gradient in update time and parallelism. Binarizing the gradient is a technique often used in hardware implementations of neural networks to reduce the complexity of computations and increase the speed of updates [10]. This is particularly relevant in

the context of our work, where the limited precision and inherent variability of memristor devices make it challenging to implement full-precision gradient updates. We compared the performance of our proposed mSGLD method with binarized gradients to a conventional SGLD method with floating-point gradients. The results are shown in Supplementary Fig. 8. Both methods show similar performance trends, with slight variations in accuracy. In addition, due to the cycle-to-cycle and device-to-device variations, it is very expensive to implement a series of ingenious modulation schemes to update conductance accurately. The mSGLD method with binarized gradients, only using a single basic writing operation SET or RESET to update conductance, can offer significant advantages in terms of update time and parallelism. It's worth noting that while binarizing the gradient can lead to faster updates and higher parallelism, it also introduces a certain level of approximation, which may impact the learning accuracy to some extent. However, our results demonstrate that the impact on accuracy is relatively minor, and the benefits in terms of speed and parallelism are significant.

Supplementary Note 6: Choosing the topology of BDNN

When choosing the topology of a neural network, several factors need to be considered, including the nature of the problem, the complexity of the data, and the desired balance between hardware complexity and model performance.

In our robot's skill learning task, the problem we were addressing required processing multi-dimensional data, specifically with 11 input dimensions. It includes the context parameters (the bowl and cup dimensions) and control parameters that the robot can choose (the axis of rotation, the cup rotation frame, and the final pitch). This necessitated a network capable of handling high-dimensional inputs, hence the first layer of our network has 11 nodes. The output layer of our network has 2 nodes, corresponding to the binary nature of our prediction task: success or failure. The outputs 01 and 10 were used to represent these two classes.

The selection of 50 nodes for the two hidden layers was based on empirical testing. Our previous work [7] also demonstrated that when used a certain number (N) of devices to represent a single weight, using more nodes for the hidden layer (K) results in a smaller KL divergence between the memristor-based outputs (Zr) and the ideal software-implemented outputs (Z). This indicates that the memristor-based output more closely approximates the ideal output, leading to lower errors and higher reliability and accuracy of the model. However, increasing the number of hidden layer's nodes would also raise the complexity and cost of the hardware. Our experimental data suggests that the cases of 50-100 nodes all have roughly equal KL values. Therefore, using 50 nodes for the two hidden layers strikes a good balance, maintaining a minimal hardware overhead while keeping the KL divergence relatively low. This represents a practical compromise that ensures quality output while managing system complexity and implementation costs. In addition, we aimed to provide the network with enough capacity to learn complex representations from the data, without overfitting. The choice of 50 nodes stroked a good balance between these objectives, providing good classification performance while keeping hardware costs manageable.

Supplementary Note 7: Technical information about the learning process of robot's skill learning task

Our mSGLD implementation is based on the preconditioned SGLD optimizer, leveraging the RMSprop optimizer available in PyTorch [11]. The integration of RMSprop with SGLD helps in navigating the BDNN's parameter space more effectively, providing a robust approach to reach convergence efficiently. The robot's pouring skill learning task used a 3D robot tabletop environment simulator, an open-source environment from the research work [12].

Regarding the training process, we set the training of our memristor BDNN for a maximum of 110 epochs. To prevent overfitting and ensure that the model generalizes well on unseen data, we incorporate an early stopping mechanism that halts training when the accuracy on the training dataset exceeds 98%. Additionally, to obtain the Gaussian conductance weight's mean and standard deviation and multiple predictions, the memristor conductance is read 5 times during each training epoch. And these readings are used for 5 separate forward propagations.

To further refine our model, we repeat the gradient calculation process 7 times for each parameter update cycle. This repetition not only increases the accuracy of the gradient estimates but also aids in identifying significant weights in the network. Weights that consistently show significant gradients across these iterations are flagged as key weights for more focused updates. This approach is crucial for the stability and performance of our BDNN, ensuring that key parameters receive the necessary attention during training.

Another important aspect of our methodology is the implementation of a reducing weight update percentage scheduler. Starting from an initial update percentage of 60%, we continuously adjust the update rate by reducing the update percentage by a factor of γ=97.5% every 2 epochs. This gradual reduction is meticulously designed to fine-tune the network by adjusting the weight update rate, facilitating a smoother transition in learning dynamics as training progresses. This step decay method is particularly effective in managing the stochasticity introduced by the memristor.

Supplementary Note 8: Impact of read-to-read variability on network performance over time

In the demonstrated robot skill learning task, we employ on-chip active learning. During the online training process, the weights are adjusted to compensate for weight deviations. The online training is a dynamic learning process where the model is trained and updated as new data is received. The weight adjustment during this process is achieved through optimization algorithms such as backpropagation and gradient descent. The model first calculates the discrepancy between the predicted and actual results (i.e., the loss function), then computes the gradients of the loss function with respect to each weight through backpropagation. These gradients describe how the weights affect the overall error, and by adjusting the weights, the error can be minimized.

However, due to device imperfections, such as device-to-device variations and read errors, weight deviations can occur, affecting the predictive performance of the model. To address this, our network model continuously adjusts the weights during the online training process to compensate for these deviations. This means that even if there are deviations in the weights initially, the model can self-correct through the online training process to achieve better predictive performance. The active learning process allows the network to continuously learn and adapt, thereby mitigating the impact of weight deviations caused by device imperfections. This can ensure that our system remains robust and reliable, even in the face of device imperfections and weight deviations.

On the other hand, we also acknowledge that after the online training process, the network weights can exhibit deviations over time due to read-to-read variations. To investigate the impact of these deviations on network performance, we have conducted simulations using a compact model developed in our previous research [13]. In this prior work, we investigated the statistical behaviors of read current noise and retention in a 1Kb filamentary memristor array. We found that the conductance distribution of different levels changes over time, and we elucidated the physical mechanism of the retention degradation.

Based on the experimental data in this prior work [13], we developed a compact model to predict the statistical conductance evolution, which can effectively evaluate the impact of read noise and retention degradation in neuromorphic computing systems. The fitting parameters are shown below:

$$\sigma(I,t) = \lambda(I)\sqrt{t} + \theta(I)$$

$$\lambda(I) = \begin{cases} 6.1\times10^{-3} & 2 \le I \le 6\mu A \\ (4.4\cdot I - 0.15)\times10^{-3} & 0.2 \le I < 2\mu A \end{cases}$$

$$\theta(I) = \begin{cases} 0.083 & 2 \le I \le 6\mu A \\ 0.046\cdot I - 0.018 & 0.2 \le I < 2\mu A \end{cases}.$$

By applying this model to our current work, we can simulate the impact of device imperfections over time on the performance of BDNN in robot skill learning task. We plotted the test set classification accuracy over time in Supplementary Fig. 10, demonstrating a relatively stable performance with accuracy levels mostly hovering around the 83% mark. The test set classification accuracy after the online training process is 82%. This illustrates the robustness of our system in handling weight deviations.

Supplementary Note 9: Energy consumption and latency estimation

We estimate the performance (energy cost and latency) of the ESCIM system and NVIDIA Tesla A100 GPU for one learning iteration in the robot skill learning task.

The network training using the mSGLD includes three phases: forward (FWD) phase, backpropagation phase (BP) and weight update phase (UPD). The FWD phase involves the flow of data from the input layer through the hidden layers to the output layer, with the aim of making a prediction. The BP phase involves calculation the gradient of weights based on the error between the predicted and desired output. And the UPD phase involves adjusting the weights value. We estimate the performance (energy consumption and latency) of the ESCIM system and NVIDIA Tesla A100 GPU for one learning iteration in the robot skill learning task. The total energy consumption (or latency) for one learning iteration is the sum of the energy consumption (or latency) of the FWD, BP and UPD phases. To ensure an equitable and direct comparison, we postulate that both GPU and memristor systems employ the identical network architecture utilized for the robot skill learning task. Each layer of the BDNN encompasses n_{weight} random normally distributed weights (Architecture of the BDNN please refer to Supplementary Table 1).

ESCIM system:

We assume that three layers of the BDNN are mapped onto the three memristor cores of the ESCIM system. The memristor cores operate in a pipeline fashion. Each memristor core comprise one $n_{in\_dim} \times n_{out\_dim}$ (see Supplementary Table 1 for details) memristor array and all the essential peripheral circuits, including drivers, ADCs, shift & adder components and activation functions (Supplementary Fig. 11).

FWD phase: The estimation of the energy cost and latency of FWD phase is referred to previous work [6]. Supplementary Fig. 11a displays the circuit modules of a memristor core. At the 130-nm technology node, the duration of the read voltage pulse is measured at 50 ns@0.2 V. The ADC block's parameters are garnered from [14], while the characteristics associated with the memristor array and other peripheral circuit components are extrapolated using the simulator. At the 28-nm technology node, the read voltage pulse duration is shorter, clocking in at 30 ns@0.2 V. We have developed and assessed a 28-nm ADC, drawing from information in [15]. All parameters, save for the driver circuits which are simulated at the 65-nm technology node, are derived from the simulated 28-nm technology node circuits. The XPEsim simulator [16] provides the typical energy cost and latency data. The detailed energy consumption and latency is listed in Extended Data Table 9, showcasing the performance of two technology nodes when subjected to an 8-bit read pulse. The energy cost and latency of the ESCIM system at the 130-nm technology node are $E_{FWD@130}$ =104.46 nJ and $T_{FWD@130}$ =0.48 μs per FWD pass, respectively. When we shift to the 28-nm technology node, the energy consumption and latency of the ESCIM system become $E_{FWD@28}$ =18.11 nJ and $T_{FWD@28}$ =0.26 μs, respectively.

BP phase: We utilized our crossbar for conductance reading to execute dot products during the FWD phase and for analog updates during the UPD phase. Nevertheless, all remaining computations, notably the gradient calculation, were exclusively carried out in CMOS. Here, given that BP can be executed with a memristor crossbar [8], we can use this as a basis for evaluating the hardware performance during the BP phase. The transpose matrix vector multiplication constitutes the most energy-intensive and time-consuming operations of the BP algorithm. When implementing BP with a memristor array, the transpose matrix vector multiplication can be accomplished with the crossbar by driving the columns and reading the rows, as depicted in Supplementary Fig. 11b. The modules required for BP phase, such as the driver, ADC, and shift & adder, are similar to those used in forward propagation. In our BDNN, the gradient calculation for the input layer memristor weight w_1, hidden layer memristor weight w_2 and output layer memristor weight w_3 is as follows:

$$\frac{\partial Loss}{\partial w_3} = a^{(2)} \cdot \varphi'^{(3)} \cdot 1$$

$$\frac{\partial Loss}{\partial w_2} = a^{(1)} \cdot \varphi'^{(2)} \cdot w_3^T \cdot \varphi'^{(3)} \cdot 1$$

$$\frac{\partial Loss}{\partial w_1} = a^{(0)} \cdot \varphi'^{(1)} \cdot w_2^T \cdot \varphi'^{(2)} \cdot w_3^T \cdot \varphi'^{(3)} \cdot 1$$

where $a^{(L)}$ is the activation of the Lth layer's neuron, and $\varphi'^{(L)}$ is the derivative of the Lth layer's activation function. $a^{(0)}$ is equal to network's input. It can be observed that, during the BP phase, only the array of hidden layer weight w_2 and output layer weight w_3 participate in the transpose matrix vector multiply computation process. We employ an evaluation process similar to FWD, using the dimensions of transpose weight w_2^T andw_3^Tas the dimensions of the weight matrix. Consequently, we obtain the energy cost and latency of the ESCIM system at the 130-nm technology node as $E_{BP@130}$ =104.00 nJ and $T_{BP@130}$ =0.45 μs, respectively, per BP pass. At the 28-nm technology node, the ESCIM system's energy cost and latency reached $E_{BP@28}$ =18.00 nJ and $T_{BP@28}$ =0.25 μs, respectively. Because in our network structure, the number of ADCs used in the BP phase (51+51) is the same as in the FWD phase (50+50+2), the energy consumption and delay of BP and FWD phases are roughly equivalent.

UPD phase: The energy consumption of the UPD phase is the sum energy consumption of the SET and RESET phases. Given that the device's conductance can be adjusted between 2.0 and 20.0 μS, an average conductance value of G_m =9.0 μS is here selected for the estimation of a modulation energy consumption. The signal SET voltage pulse widths are 50 ns@2.0 V and 40 ns@2.0 V at the 130- and 28-nm technology nodes, respectively. And RESET widths are 50 ns@1.95 V and 40 ns@1.6 V, respectively. Furthermore, our proposed mSGLD with a scheduler reducing the weight update percentage will also reduce the quantity of weight updates. As the weight update

percentage continues to decline with the number of training iterations and one out of three devices is modulated in a weight, we presume the average device update percentage to be 50%×0.333=16.67% for generalization. Therefore, the energy consumption during the UPD phase can be calculated as follows:

$$E_{UPD} = (V_{SET}^2 + V_{RESET}^2) \cdot G_m \cdot PulseWidth \times (N_{weight} \cdot 16.67\%).$$

For the calculation of delay in the UPD phase, we posit that the modulation of conductance is executed in a parallel manner row by row, with the SET and RESET operations being carried out consecutively for one update iteration in a memristor core. The maximum number of rows in three cores is 51, hence, the delay during the weight update phase can be computed as:

$$T_{UPD} = PulseWidth \times 51 \times 2$$

Hence, the energy cost and latency of the ESCIM system at the 130-nm technology node are $E_{UPD@130}$ =5.71 nJ and $T_{UPD@130}$ =5.10 μs, respectively, in UPD phase. At the 28-nm technology node, the energy cost and latency of the ESCIM system reached $E_{UPD@28}$ =3.84 nJ and $T_{UPD@28}$ =4.08 μs, respectively.

Summary and discussion: Finally, by summing the energy consumption of the FWD, BP and UPD phases, we obtain the energy consumptions of 130-nm and 28-nm ESCIM system for one learning iteration are 214.17 nJ and 38.98 nJ, respectively. And the latencies are 6.04 μs and 4.59 μs, respectively. The energy consumption and delay of each phase, as well as the overall summary, are presented in Extended Data Table 9.

Here, we discuss the impact of TIA, DAC, and ADC on the energy and speed of the ESCIM system. These components indeed consume a significant portion of energy in memristor in-memory computing systems. Since the input value of the layer is encoded by eight sequential pulses based on its quantized bit number, DACs are not necessary for the network, instead, shift adders are used to shift and sum the sensed results. Given that only one SET (or RESET) pulse is applied to the selected device to increase (or decrease) the conductance without verifying whether it has reached the target value or not, TIAs are also not needed. The energy breakdown of the memristor chip during the learning process is presented in Extended Data Fig. 10. We discuss the 130nm case as an example here. The primary energy consumption stems from the ADC (203.89 nJ), accounting for 95.2% of the network's total energy usage. This energy usage could be further minimized by refining the ADC design. The remaining energy consumption is attributed to the memristor array and other circuit modules, such as line drivers and shift adders. Regarding latency, the most significant delay arises from the device update phase due to the parallel modulation approach implemented row by row. This delay could be further diminished by employing high-parallel modulation methods.

NVIDIA A100 GPU-based system:

We also estimated the energy consumption and computing latency of a typical CMOS-based digital computing platform NVIDIA Tesla A100 GPU. This computing system generally consists of processors and a main memory. For simplification, we assume that the A100 GPU operate at the peak random number generation throughput, i.e., the peak operations per second (peak OPS, OPS_{GPU}=19.5 TOPS), and thermal design power (TDP, P_{GPU}=400 W) (please refer to Supplementary Table 2).

<u>*FWD phase:*</u> To accomplish a FWD phase of the BDNN, the GPU should generate a normally distributed weight sample and then perform VMM operations layer by layer. To generate weight sample, the GPU first reads the mean and standard deviation parameters of a layer from main memory. Hence, the latency of reading parameters for a layer is:

$$T_{read} = T_{GPU} \times 2$$

In relation to the sample and VMM steps, we take into account the GPU relative occupancy O_{sample} and O_{VMM} values, respectively, to accurately estimate the energy expenditure of a layer. The occupancy in the sample step, O_{sample}, is derived from [17] based on the number of samples n_{sample} for a layer. It's important to note that the relative occupancy of [17] is grounded on uniformly distributed pseudorandom number generation, which could have a lower occupancy than that of normally distributed pseudorandom number generation. Consequently, the O_{sample} of three layers are each 30%. The average occupancy of the VMM step, O_{VMM}, can be determined by the number of weights n_{weight} in a layer and cores in the GPU N_{core}:

$$O_{VMM} = \frac{\frac{n_{weight}}{N_{core}}}{\left\lceil \frac{n_{weight}}{N_{core}} \right\rceil}$$

where $\lceil x \rceil$ represents the ceiling function. Therefore, the O_{VMM} of the three layers are 17.36%, 73.78% and 2.95%, respectively.

Then, according to the given sampling throughput with a normal distribution (S_{GPU}=236.6 GSamples/sec), the estimated energy cost and latency of the sample step for a layer can be calculated as:

$$T_{sample} = \frac{n_{sample}}{S_{GPU}}$$

$$E_{sample} = T_{sample} * P_{GPU} * O_{sample}$$

Based on the given peak OPS (OPS_{GPU}=19.5 TOPS), the estimated energy cost and latency of the VMM operation step for a layer can be calculated as:

$$T_{VMM} = \frac{n_{\mathrm{multiplication}} + n_{\mathrm{addition}}}{OPS_{GPU}}$$

$$E_{VMM} = T_{VMM} * P_{GPU} * O_{VMM}$$

By accumulating the energy cost and latency of three layer, we can obtain the computational cost in performing a FWD pass as listed in Supplementary Table 3.

BP phase: During the BP phase, only the array of hidden layer weight w_2 and output layer weight w_3 participate in the transpose matrix vector multiply computation process. Hence, the BP phase primarily engages the network's final two layer weights. As the GPU only needs to read out the weight sample values from the FWD phase when calculating the gradient, the read time for the two-layer network is: $T_{read} = T_{GPU} \times 2$. Additionally, the VMM operations of gradient calculation in the BP phase necessitate the same MAC number as the FWD phase of the last two layers. Therefore, the energy cost and delay of the BP phase can be determined as outlined in Supplementary Table 3.

UPD phase: During the UPD phase of SGLD, the GPU produces Gaussian distributed random numbers as additive noise for the gradient, reads mean parameters and standard deviation parameters, and executes a MAC operation to acquire the update for each Gaussian weight. Consequently, each weight entails a MAC operation and a random number generation, mirroring the circumstances of the FWD phase. Given that the network updates both mean parameters and standard deviation parameters, and the GPU finally writes the updated parameter to the main memory, the energy expenditure and delay of the UPD phase is twice that of the FWD phase, as detailed in Supplementary Table 3. For completeness, it should be noted that the energy costs of the writing operations required to update all the weight parameters in memory is still excluded in this estimation.

Summary of the comparison with GPU: When contrasted with the NVIDIA Tesla A100 GPU, the energy expenditure of the memristor-based ESCIM system is roughly 28 times more efficient at 130 nm and 153 times more efficient at 28 nm. Regarding latency, the ESCIM system's latency improves by 26% at 130 nm and 44% at 28 nm compared to the GPU's. It's crucial to emphasize that the NVIDIA Tesla A100 GPU uses 7-nm CMOS technology, which is significantly more aggressively downscaled than the nodes employed in our ESCIM system.

Supplementary Video

Caption for Supplementary Video 1

The video shows the visualization results for 3 different cases (bowl and cup pairs), including small green bowl and yellow cup, yellow bowl and red cup, and blue bowl and large orange cup. The visualization results for different amounts of newly-added highest-uncertainty data shown that BDNN's classification performance is enhanced by highest-uncertainty data. The visualizations of pouring result shown the robot is pouring the beads from the cup into the bowl.

References

[1] Nvidia ampere architecture whitepaper: Nvidia A100 tensor core GPU architecture. https://images.nvidia.com/aem-dam/en-zz/Solutions/data-center/nvidia-amperearchitecture-whitepaper.pdf , accessed: 2022-03-08

[2] How GPU Computing Works, Stephen Jones, Nvidia. https://www.nvidia.com/enus/on-demand/session/gtcspring21-s31151/ , accessed: 2022-03-08

[3] Nvidia curand: Random number generation on nvidia gpus. https://developer.nvidia.com/curand , accessed: 2022-03-08

[4] Ambrogio, S. et al. Statistical Fluctuations in HfO_x Resistive-Switching Memory: Part II—Random Telegraph Noise. IEEE Trans. Electron Devices 61, 2920–2927 (2014).

[5] Puglisi, F. M., Pavan, P. & Larcher, L. Random telegraph noise in HfOx Resistive Random Access Memory: From physics to compact modeling. in 2016 IEEE International Reliability Physics Symposium (IRPS) MY-8-1-MY-8-5 (IEEE, 2016). doi:10.1109/IRPS.2016.7574624.

[6] Lin, Y., Zhang, Q., Gao, B. et al. Uncertainty quantification via a memristor Bayesian deep neural network for risk-sensitive reinforcement learning. Nat Mach Intell 5, 714–723 (2023).

[7] Lin, Y. et al. Bayesian Neural Network Realization by Exploiting Inherent Stochastic Characteristics of Analog RRAM. in 2019 IEEE International Electron Devices Meeting (IEDM) 14.6.1-14.6.4 (IEEE, San Francisco, CA, USA, 2019). doi:10.1109/IEDM19573.2019.8993616.

[8] Hasan, R. & Taha, T. M. Enabling back propagation training of memristor crossbar neuromorphic processors. in 2014 International Joint Conference on Neural Networks (IJCNN) 21–28 (2014).

[9] Kampffmeyer, M., Salberg, A.-B. & Jenssen, R. Semantic Segmentation of Small Objects and Modeling of Uncertainty in Urban Remote Sensing Images Using Deep Convolutional Neural Networks. in 2016 IEEE Conference on Computer Vision and Pattern Recognition Workshops (CVPRW) 680–688 (2016).

[10] Zhang, Q. et al. Sign backpropagation: An on-chip learning algorithm for analog RRAM neuromorphic computing systems. Neural Networks 108, 217–223 (2018).

[11] Welling, M. & Teh, Y. W. Bayesian learning via stochastic gradient Langevin dynamics. in Proceedings of the 28th international conference on machine learning (ICML-11) 681–688 (Citeseer, 2011).

[12] Wang, Z., Garrett, C. R., Kaelbling, L. P. & Lozano-Pérez, T. Learning

compositional models of robot skills for task and motion planning. The International Journal of Robotics Research 40, 866–894 (2021).

[13] Zhao, M. et al. Investigation of statistical retention of filamentary analog RRAM for neuromophic computing. in 2017 IEEE International Electron Devices Meeting (IEDM) 39.4.1-39.4.4 (IEEE, San Francisco, CA, USA, 2017).

[14] S. M. Louwsma, J. M. van Tuijl, M. Vertregt and B. Nauta, "A 1.35 GS/s, 10b, 175 mW time-interleaved AD converter in 0.13 μm CMOS," 2007 IEEE Symposium on VLSI Circuits, 2007, pp. 62-63, doi: 10.1109/VLSIC.2007.4342766.

[15] B. R. Gregoire and U. Moon, "An Over -60 dB True Rail-to-Rail Performance Using Correlated Level Shifting and an Opamp With Only 30 dB Loop Gain," in IEEE Journal of Solid-State Circuits, vol. 43, no. 12, pp. 2620-2630, Dec. 2008, doi: 10.1109/JSSC.2008.2006312.

[16] Zhang, W. et al. Design guidelines of RRAM-based neural-processing unit: a joint device– circuit–algorithm analysis. In 2019 56th ACM/IEEE Design Automation Conference (DAC) 63.1 (IEEE, 2019).

[17] Hasan, R. & Taha, T. M. Enabling back propagation training of memristor crossbar neuromorphic processors. in 2014 International Joint Conference on Neural Networks (IJCNN) 21–28 (2014).
